# The mitochondrial genome of Muga silkworm (*Antheraea assamensis*) and its comparative analysis with other lepidopteran insects

**DOI:** 10.1371/journal.pone.0188077

**Published:** 2017-11-15

**Authors:** Deepika Singh, Debajyoti Kabiraj, Pragya Sharma, Hasnahana Chetia, Ponnala Vimal Mosahari, Kartik Neog, Utpal Bora

**Affiliations:** 1 Bioengineering Research Laboratory, Department of Biosciences and Bioengineering, Indian Institute of Technology Guwahati, Assam, India; 2 Centre for the Environment, Indian Institute of Technology Guwahati, Assam, India; 3 Department of Bioengineering and Technology, Gauhati University Institute of Science and Technology (GUIST), Gauhati University, Guwahati, Assam, India; 4 Biotechnology Section, Central Muga Eri Research & Training Institute (CMER&TI), Lahdoigarh, Jorhat, Assam, India; Natural Resources Canada, CANADA

## Abstract

Muga (*Antheraea assamensis*) is an economically important silkmoth endemic to the states of Assam and Meghalaya in India and is the producer of the strongest known commercial silk. However, there is a scarcity of genomic and proteomic data for understanding the organism at a molecular level. Our present study is on decoding the complete mitochondrial genome (mitogenome) of *A*. *assamensis* using next generation sequencing technology and comparing it with other available lepidopteran mitogenomes. Mitogenome of *A*. *assamensis* is an AT rich circular molecule of 15,272 bp (A+T content ~80.2%). It contains 37 genes comprising of 13 protein coding genes (PCGs), 22 tRNA and 2 rRNA genes along with a 328 bp long control region. Its typical *tRNA*^*Met*^*-tRNA*^*Ile*^*-tRNA*^*Gln*^ arrangement differed from ancestral insects (*tRNA*^*Ile*^*-tRNA*^*Gln*^*-tRNA*^*Met*^). Two PCGs *cox1* and *cox2* were found to have CGA and GTG as start codons, respectively as reported in some lepidopterans. Interestingly, *nad4l* gene showed higher transversion mutations at intra-species than inter-species level. All PCGs evolved under strong purifying selection with highest evolutionary rates observed for *atp8* gene while lowest for *cox1* gene. We observed the typical clover-leaf shaped secondary structures of tRNAs with a few exceptions in case of *tRNA*^*Ser1*^ and *tRNA*^*Tyr*^ where stable DHU and TΨC loop were absent. A significant number of mismatches (35) were found to spread over 19 tRNA structures. The control region of mitogenome contained a six bp (CTTAGA/G) deletion atypical of other *Antheraea* species and lacked tandem repeats. Phylogenetic position of *A*. *assamensis* was consistent with the traditional taxonomic classification of Saturniidae. The complete annotated mitogenome is available in GenBank (Accession No. KU379695). To the best of our knowledge, this is the first report on complete mitogenome of *A*. *assamensis*.

## Introduction

Mitochondria are known to have descended from α-proteobacterium endosymbionts and have retained numerous bacterial features [1]. Apart from being the powerhouse of the cell, they are involved in various cellular processes like fatty acid metabolism, apoptosis and aging [2]. These functions are carried out by many nuclear-encoded genes along with extra-nuclear genes that include protein-coding genes (PCGs), transfer RNA (tRNA) and ribosomal rRNA (rRNA) genes and are present in the mitogenome which is mostly circular and self-replicative. It also consists of several non-coding regions with the longest being AT-rich control region comprising of several conserved regions and repeats. Although small in size, the mitogenome governs maternal inheritance and has several unique features like faster evolution rate, low or absence of homologous recombination, evolutionary conserved gene products and richness in genetic polymorphism. This makes it a potential marker for barcoding, phylogeographic and phylogenetic studies [3]. It plays a potential role in molecular evolutionary studies by elucidating evolutionary models and substitution patterns that vary timely and across sequences. Compared to individual genes, whole mitogenomes are more informative phylogenetic models due to its multiple genome level features like gene position, content, secondary structures of RNA and control region.

Over the past few decades, animal mitogenomes, particularly insects (~80% of the sequenced arthropods), have been widely studied for comparative genomics and molecular systematics [3–4]. More than 250 Lepidopteran insect mitogenomes have been sequenced till now using Sanger sequencing and next generation sequencing (NGS) technologies as found in GenBank (https://www.ncbi.nlm.nih.gov/). The mitogenome ranges from 14–16 kilo basepairs (kb) in majority of the lepidopterans and consists of 37 genes (13 PCGs, 2 rRNA genes, 22 tRNA genes) along with a control region. The PCGs encode 2 subunits of ATPase (ATP6 and ATP8), 3 subunits of cytochrome *c* oxidase (COI, COII and COIII), 1 subunit of cytochrome *b* (CYTB) and 7 subunits of NADH dehydrogenase (ND1, ND2, ND3, ND4, ND4L, ND5 and ND6). These proteins are responsible for oxidative phosphorylation (OXPHOS) as they form essential mitochondrial membrane-associated protein complex systems [3]. The control region plays a role in the replication and transcription of mitogenome. The utilities and potential of mitochondrial PCGs (*cox1* and *cox2*) as barcode markers have been well demonstrated in the order Lepidoptera [5]. The comparative mitogenome analysis also elucidated sequence divergence patterns among domesticated and non-domesticated lepidopteran mitogenomes [6–7]. Although frequency of mitogenome sequencing of lepidopterans has increased, the evolutionary relationships among many family members of the same order have been hardly investigated [8–9].

Saturniidae is the most diverse family of wild silkmoths which include giant moths, royal moths and emperor moths. Most of these silkmoths are unexplored and might have potential significance in sericulture [10]. Till date, mitogenome data of eleven species have been sequenced and made available in GenBank. However, numerous individual gene sequences of various wild species are also available whose complete mitogenome sequencing has not been done. *Antheraea assamensis*, muga silkmoth is a semi-domesticated and one of the economically important moths of the Saturniidae family. It is endemic to erstwhile undivided Assam and its adjoining hilly areas located in the North-Eastern part of India. It is a multivoltine and polyphagous moth that primarily thrives on two main host plants *Persea bombycina* and *Litsea monopetala* [11]. Its silk has wide applications in textile industry and has great potential as a biomaterial due to its unique biophysical properties like golden lustre, tenacity and absorption of UV radiation [12]. Like other *Antheraea* species, it produces reelable silk which is the most expensive silks of the world. However, its semi-domesticated nature and extraction of fibroin directly from cocoon fibers limit its extensive rearing and prospects of global applicability as a biomaterial. The whole genome of *A*. *assamensis* is not yet available. However, *de novo* transcriptome data from our laboratory is available in GenBank (Accession Number- SRX1293136, SRX1293137 and SRX1293138) [13].

In the present study, we report the whole mitogenome sequence of *A*. *assamensis* using NGS and comparative analysis of its sequences and genome architectures with that of the other lepidopterans. The comparative study was based on several characteristics such as genome arrangement, PCGs, tRNAs, rRNAs, nucleotide composition, codon usage, evolutionary rates, gene divergence, conserved regions in control region, etc. Furthermore, phylogenetic trees inferred using datasets like nucleotide sequences of 13 PCGs and 13 PCGs+2 rRNAs were analyzed to elucidate the relationships among lepidopteran insects. This study will facilitate a better understanding of the comparative and evolutionary biology of *A*. *assamensis* with the other lepidopteran insects.

## Materials and methods

### Sample processing, DNA sequencing and assembly

**Fig 1:**
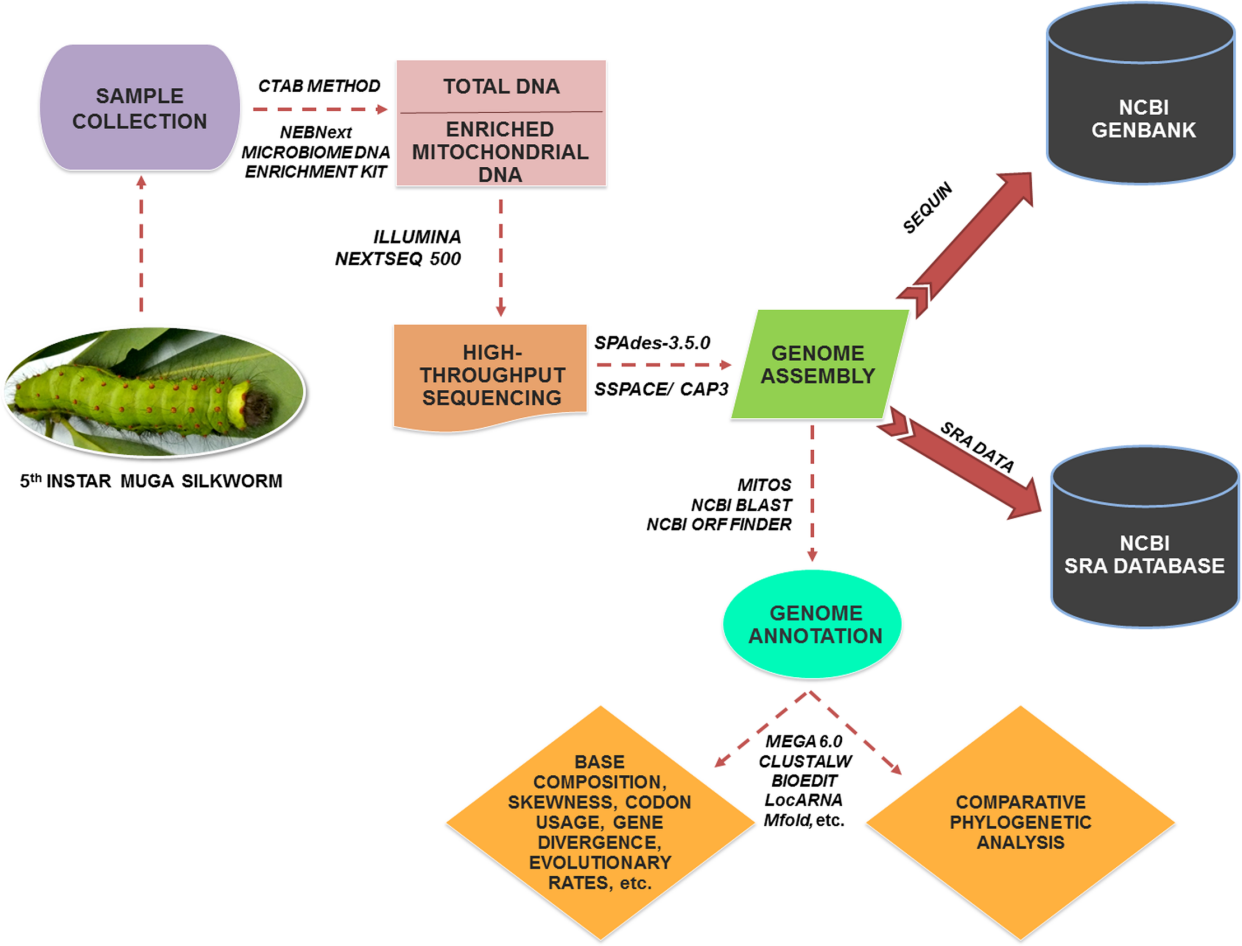
A pictorial overview of the methodologies used for sequencing and analysis of *A*. *assamensis* mitogenome.

The larvae of *A*. *assamensis* were reared on *Persea bombycina* or Som (a primary host plant) in the experimental field of Central Muga Eri Research and Training Institute, Lahdoigarh, Jorhat, Assam, India following recommended package of practices from brushing till attainment of maturity [14]. The effective rate of rearing of silkworms referred as the number of mature larvae collected from the total brushed larvae was found to be 61.63%. Other rearing parameters like body weight of mature larvae (11.84 g), cocoon weight (6.26 g) and shell weight (0.55 g) were found to be suitable for experimental work. The fifth instar larval stage of *A*. *assamensis* was used for the present study (Sample ID: CMERI-Aa-001). The larvae were sterilized by washing with 70% ethanol before being processed for mitogenome studies. The larvae were stored in 95% absolute ethanol at -80°C for future use. The total DNA was extracted using CTAB (Cetyl trimethylammonium bromide) based buffer and silica column by the service provider (Genotypic Technology Pvt. Ltd. Bengaluru, India). Subsequently, mitochondrial DNA was enriched from total DNA extracted once the integrity, quantity and purity of extracted DNA was confirmed by agarose gel electrophoresis, light absorbance and fluorescence spectroscopy. The complete overview of sequencing and analysis of mitochondrial genome of *A*. *assamensis* is represented in Fig 1.

Briefly, the preferential enrichment of mitochondrial DNA was carried out with NEBNext microbiome DNA enrichment kit (New England Biolabs, USA) which selectively removes CpG-methylated eukaryotic nuclear DNA. The enriched mitochondrial DNA was subjected to DNA sequencing at Genotypic Technology’s Genomics facility where the enriched DNA was acoustically sheared to 300–500 bp using a specially designed Covaris microTube (for focused ultra-sonication). The fragmented mitochondrial DNA was cleaned up using HighPrep beads (MagBio Genomics, Inc, Gaithersburg, Maryland) and subjected to end-repair, A-tailing and ligation with multiplex adaptors using the NEXTFlex DNA Sequencing kit (Catalogue # 5140–02, Bioo Scientific). The ligated DNA was cleaned with HighPrep beads and subjected to amplification via PCR as follows: initial denaturation at 98˚C for 2 min; 10 cycles of denaturation at 98˚C for 30 sec, annealing at 65˚C for 30 sec and extension at 72˚C for 60 sec; and a final extension at 72˚C for 4 min) using the primers provided by NEXTFlex DNA Sequencing kit. Finally, the PCR product was purified with HighPrep beads, quantified and fragment range was assessed using Qubit fluorometer and the Agilent D1000 Tape (Agilent Technologies), respectively prior to sequencing. The sequencing was carried out in Illumina NextSeq 500 sequencer (Illumina Inc, Sandiego, USA) through 2 × 150 bp paired-end chemistry. The raw paired end data obtained was de-multiplexed using Bcl2fastQ, assessed with FastQC tool [15] to remove low quality bases (Q<30) and preprocessed using ABLT-Scripts (Genotypic technology, Bangalore India). The SPAdes-3.5.0 was used for sequence assembly followed by the filling of gaps. Finally, scaffolding of assembled contigs and clustering were carried out with SSPACE and CAP3 programs, respectively [16–17].

### Genome annotation, visualization and comparative analysis

The assembled scaffolds were annotated using MITOS web-server to predict the location of protein coding regions/genes, tRNAs, rRNAs and secondary structures of tRNAs. MITOS is a widely used webserver for the annotation of metazoan mitochondrial genomes due to its advanced annotation methodology [18]. The location of PCGs, tRNAs and rRNAs was further confirmed using tools like NCBI (National Center for Biotechnology Information)–BLAST, BioEdit and Clustal Omega by comparing the sequences of *A*. *assamensis* with the respective sequences published for other lepidopteran insects [19–20]. The boundaries of PCGs i.e. initiation and termination codons were determined using NCBI ORF Finder (http://www.ncbi.nlm.nih.gov/gorf/gorf.html) by specifying the invertebrate mitochondrial genetic code. Further, the number of overlapping or spacer regions between the genes were visualized and calculated manually. The control region was validated by comparing with the available sequences in GenBank and the tandem repeats in this region were searched through Tandem Repeat Finder [20–21]. Then, the whole mito-map was constructed and visualized using Blast Ring Image Generator (BRIG) tool [22]. Finally, the complete annotated file of mitochondrial genome was prepared using NCBI Sequin tool (http://www.ncbi.nlm.nih.gov/Sequin/) and the sequin file along with SRA data were submitted to NCBI GenBank.

For understanding evolutionary relationships of *A*. *assamensis* with various lepidopterans as listed in Table 1, the sequences of whole mitogenome, coding regions, tRNAs, rRNAs and control region were retrieved from the NCBI GenBank database and a comparative analysis was performed.

**Table 1:** List of species considered for comparative mitogenome study. (Highlighted ones are the organisms of Bombycoidea superfamily used for comparative mitogenome analysis with respect to *A*. *assamensis*).

Superfamily	Family	Species	Size (bp)	Accession No.	References
Bombycoidea	Saturniidae	***Antheraea assamensis***	15,272	KU379695	**This study**
		*Attacus atlas*	15,282	KF006326	[23]
		*Samia cynthia ricini*	15,384	JN215366	[24]
		*Antheraea pernyi*	15,575	AY242996	[25]
		*Antheraea yamamai*	15,338	EU726630	[26]
		*Antheraea frithi*	15,338	NC_027071	GenBank
		*Actias selene*	15,236	NC_018133	[27]
		*Saturnia boisduvalii*	15,360	NC_010613	[28]
		*Eriogyna pyretorum*	15,327	FJ685653	[29]
	Bombycidae	*Bombyx mandarina*	15,682	AY301620	[30]
		*Bombyx mori*	15,656	AB070264	[31]
		*Rondotia menciana*	15,301	KC881286	[32]
	Sphingidae	*Manduca sexta*	15,516	EU286785	[33]
		*Sphinx morio*	15,299	KC470083	[34]
Geometroidea	Geometridae	*Biston panterinaria*	15,517	NC_020004	[35]
		*Phthonandria atrilineata*	15,499	NC_010522	[36]
Cossoidea	Cossidae	*Eogystia hippophaecolus*	15,431	KC831443	[37]
Papilionoidea	Riodinidae	*Abisara fylloides*	15,301	HQ259069	[38]
	Lycaenidae	*Spindasis takanonis*	15,349	HQ184266	[39]
		*Protantigius superans*	15,248	HQ184265	[39]
	Papilionidae	*Papilio maraho*	16,094	FJ810212	[40]
		*Parnassius bremeri*	15,389	NC_014053	[41]
	Nymphalidae	*Danaus plexippus*	15,314	NC_021452	GenBank
		*Eumenis autonoe*	15,489	GQ868707	[42]
Noctuoidea	Lymantriidae	*Gynaephora menyuanensis*	15,770	KC185412	[43]
		*Lymantria dispar*	15,569	NC_012893	GenBank
Yponomeutoidea	Plutellidae	*Plutella xylostella*	16,014	KM023645	[44]
Tortricoidea	Tortricidae	*Adoxophyes honmai*	15,680	DQ073916	[45]
		*Cydia pomonella*	15,253	JX407107	[46]
Pyraloidea	Crambidae	*Ostrinia furnacalis*	14,536	NC_003368	[47]
		*Ostrinia nubilalis*	14,535	NC_003367	[47]
Diptera		*Drosophila melanogaster*	19,517	NC_001709	[48]
(Outgroup)		*Drosophila yakuba*	16,019	NC_001322	[49]
		*Anopheles gambiae*	15,363	NC_002084	[50]

#### Comparative mitogenome analysis

Comparative mitogenome analysis was carried out in order to find out similarities and differences of *A*. *assamensis* with other insects in terms of characteristics like genome size, organization, structure, gene content and rearrangement. The sequence homology of *A*. *assamensis* mitogenome with selected insects was also performed using Clustal Omega [20].

#### Comparative analysis among protein coding genes (PCGs)

The PCGs of *A*. *assamensis* were compared with selected organisms based on their number, length, initiation and termination codons. The sequence similarity among the PCGs was determined by aligning the sequences of *A*. *assamensis* with selected Bombycoids using Clustal Omega. MEGA 6.0 tool was used for determining the gene-by-gene divergences in 13 PCGs in terms of phylogenetically informative sites, conserved sites as well as variable sites [51]. The same tool was used for estimating nucleotide substitution and evolutionary rates among the genes in terms of transitions (ts), transversions (tv), rate of synonymous substitutions (Ks), rate of non-synonymous substitutions (Ka) and their ratios. Further, the correlation between GC content and Ka/Ks ratio was studied in order to predict the effect of GC content on evolutionary rates of PCGs.

#### Comparative analysis among transfer RNAs (tRNAs)

The length, arrangement, secondary structures and variation in the structures were studied among the selected organisms. Homologous sites in their secondary structures were identified by aligning the tRNA sequences using LocARNA tool and the type/number of mismatches were calculated [52].

#### Comparative analysis among ribosomal RNAs (rRNAs)

The number, type, location and length of rRNAs in *A*. *assamensis* were compared with the selected organisms in order to study the conservation pattern. In addition, sequence homology of rRNAs of *A*. *assamensis* with the selected organisms was determined and gene-by-gene divergences among the rRNAs were studied using MEGA 6.0 tool [51].

#### Comparative analysis among control region (A+T rich region)

The control region of *A*. *assamensis* was compared with selected organisms based on the length and location. Multiple sequence alignment of control region was then carried out using Clustal Omega and their conserved regions, repeats and indels were visualized using BioEdit tool [19].

#### Comparative analysis among overlapping sequence (OS) and intergenic spacer (IGS) regions

The OS and IGS regions of *A*. *assamensis* mitogenome were compared with selected organisms (Table 1) in terms of number, length and location. The sequence homology of OS and IGS was determined through sequence alignment using Clustal Omega and BioEdit tools to find conserved motifs.

#### Comparative analysis with respect to nucleotide composition, skewness and codon usage

The nucleotide composition of sequences of whole mitochondrial genome, concatenated and individual PCGs, tRNAs, rRNAs, spacers and control region was calculated using MEGA 6.0 software [51]. The base composition skewness was also calculated for all the regions of mitogenome using the formula (Eq 1 and Eq 2) described by Junqueira et al., 2004 [53].

ATskew=(A−T)/(A+T) (1)

GCskew=(G−C)/(G+C) (2)

Where A, T, G and C denote the frequencies of respective bases.

The codon usage and relative synonymous codon usage (RSCU) values of PCGs were determined using MEGA 6.0.

### Comparative phylogenetic analysis

About 34 organisms representing 13 different families of 8 super families within the order Lepidoptera were considered for phylogenetic analysis along with three organisms belonging to the order Diptera as outgroups (Table 1). The phylogenetic relationship of *A*. *assamensis* was elucidated using two criteria’s: (i) concatenated nucleotide sequences of 13 PCGs and (ii) concatenated nucleotide sequences of 13 PCGs and 2 rRNAs.

The nucleotide sequences of each PCG were translated via amino acid alignment using MAFFT algorithm in TranslatorX server which were then back translated into nucleotide alignment with GBlocks [54]. The individual nucleotide sequences obtained from GBlocks and rRNA sequences were then concatenated for further phylogenetic analysis. Substitution model optimization for each dataset was performed in jModelTest 2.1.7 [55–56]. The GTR+G+I model was selected as the best substitution model for both the datasets to conduct maximum likelihood (ML) and bayesian inference (BI) analysis. Phylogenetic trees for both the datasets were inferred using maximum likelihood (ML) method implemented in RaxmlGUI v1.3 with 1000 bootstrap replicates [57]. The BI analysis was conducted using Markov chain Monte Carlo (MCMC) method in MrBayes v3.2.6 for 1,150,000 and 1,320,000 generations for PCG and PCG+rRNA datasets, respectively [58–59]. Trees were sampled every 1000 generations and the first 25% were discarded as burn-in. The stationary was considered to be reached when the average standard deviation of split frequency reached below 0.01. Other parameters like effective sample size (ESS) and potential scale reduction factor (PSRF) were evaluated for stationary using Tracer v1.6 [60]. The consensus phylogenetic trees obtained for both the datasets were visualized using iToL v3.6.1 tool [61].

## Results and discussion

### Sample processing, DNA sequencing and assembly

Total DNA extracted from fifth instar larvae of *A*. *assamensis* was found to be optimal (1363.23 ng/μl concentration by NanoDrop spectrophotometer and 234.36 ng/μl concentration by Qubit fluorometer) for mitochondrial DNA enrichment. The mitochondrial DNA was enriched from total DNA for library preparation. The library profile showed that size of mitogenome fragments were in the range of 180 to 880 bp. However, total insert size distribution was from 300 to 1000 bp as it involved ~120 bp adapters along with mitogenome fragments. In addition to the proper distribution of fragments, their concentrations (~27.2 ng/μl) were also found to be adequate for sequencing (S1 Fig). The sequencing resulted in 32,12,599 total number of raw reads, out of which around 28,69,153 high quality reads were processed for scaffold preparation. These reads were used for mitogenome scaffold preparation through *de novo* assembly of sequenced contigs using SPAdes-3.5.0, SSPACE and CAP3 program. Finally, a scaffold of 15,272 bp length was obtained which represents the whole mitogenome sequence of *A*. *assamensis*.

### Genome annotation, visualization and comparative analysis

**Fig 2:**
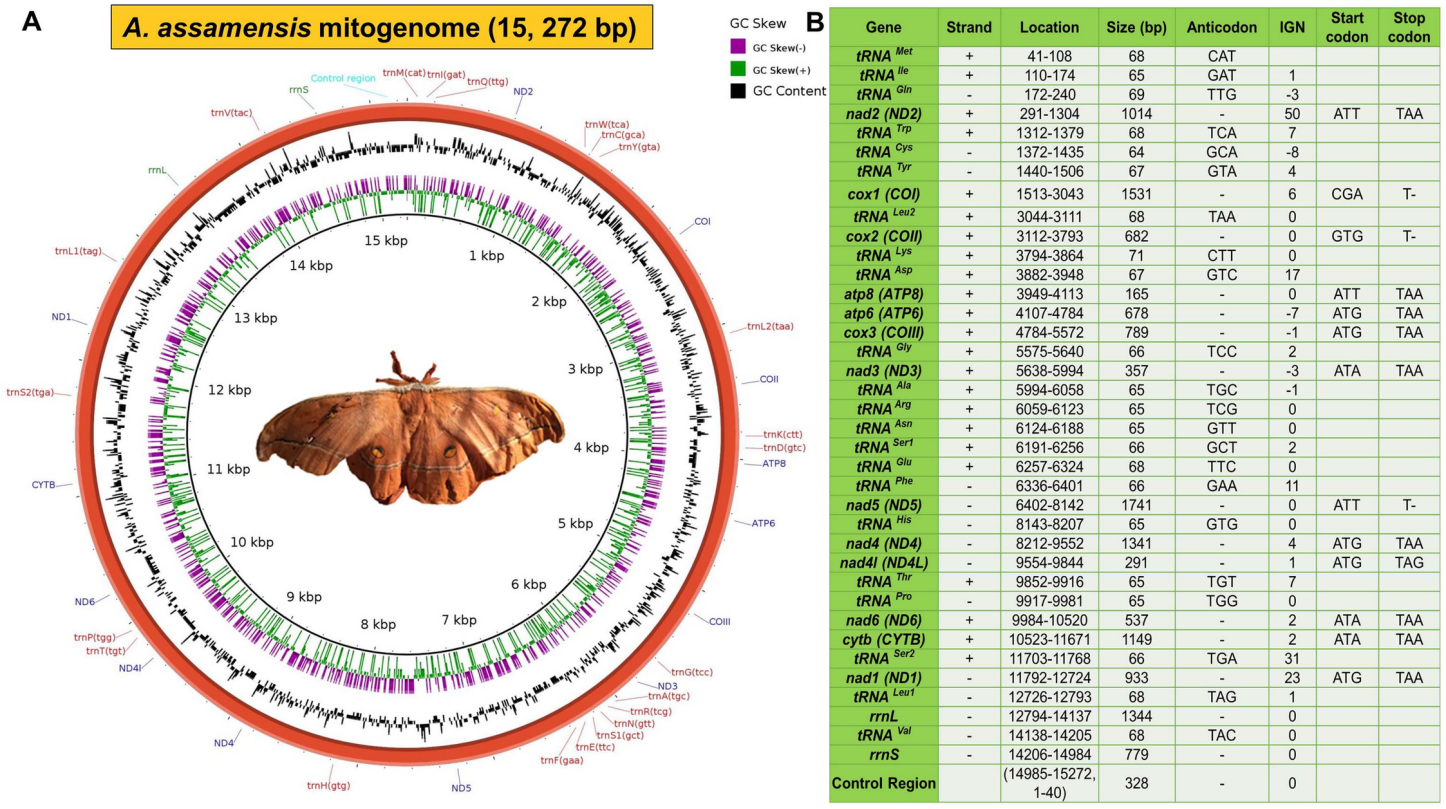
*A*. *assamensis* mitochondrial genome. **(A) Circular map (B) Characteristic features of the mitogenome.** *tRNAs* are represented as *trn* followed by the IUPAC-IUB single letter amino acid codes e.g. *trnM* denote *tRNA*^*Met*^. IGN represents (+) values as intergenic nucleotides and (-) values as overlapping regions.

The annotated mitochondrial genome of *A*. *assamensis* appeared to be a closed circular structure comprising of 37 genes (13 PCGs, 22 tRNAs and 2 rRNAs) along with a non-coding control region over its 15,272 bp length. 23 genes were found to be encoded by the majority strand or J-strand (+) and the remaining 14 genes by the minority strand or N-strand (-). The whole mito-map constructed using BRIG along with its characteristics like gene location and arrangement are shown in Fig 2. The full annotated mitogenome sequence and SRA data of *A*. *assamensis* submitted to NCBI GenBank are available under the accession numbers KU379695 and SRR3948351, respectively.

#### Comparative mitogenome analysis

In order to study the conservation pattern, features like genome size, gene content, genome organization, structure, rearrangement and sequence of *A*. *assamensis* mitogenome were compared with other lepidopterans belonging to various levels of taxonomic classification (Table 1).

We found that the length of *A*. *assamensis* mitogenome was smaller than that of other sequenced Bombycoids and just bigger than that of *Actias selene* (15,236 bp) and *A*. *artemis* (15,243 bp) [27, 62]. Notably, it lies within the characteristic size of most of the insects (14 to 20 kb). In insects, mitogenome size variation may be attributed to variation in non-coding regions especially the control region that shows great differences in length as well as pattern. It collectively leads to higher degree of gene rearrangements. In contrast, PCGs are quite stable in the mitogenome [23–24]. Smaller mitogenome size in *A*. *assamensis* may be favored to reduce accumulation of slightly deleterious mutations during its evolution.

**Fig 3:**
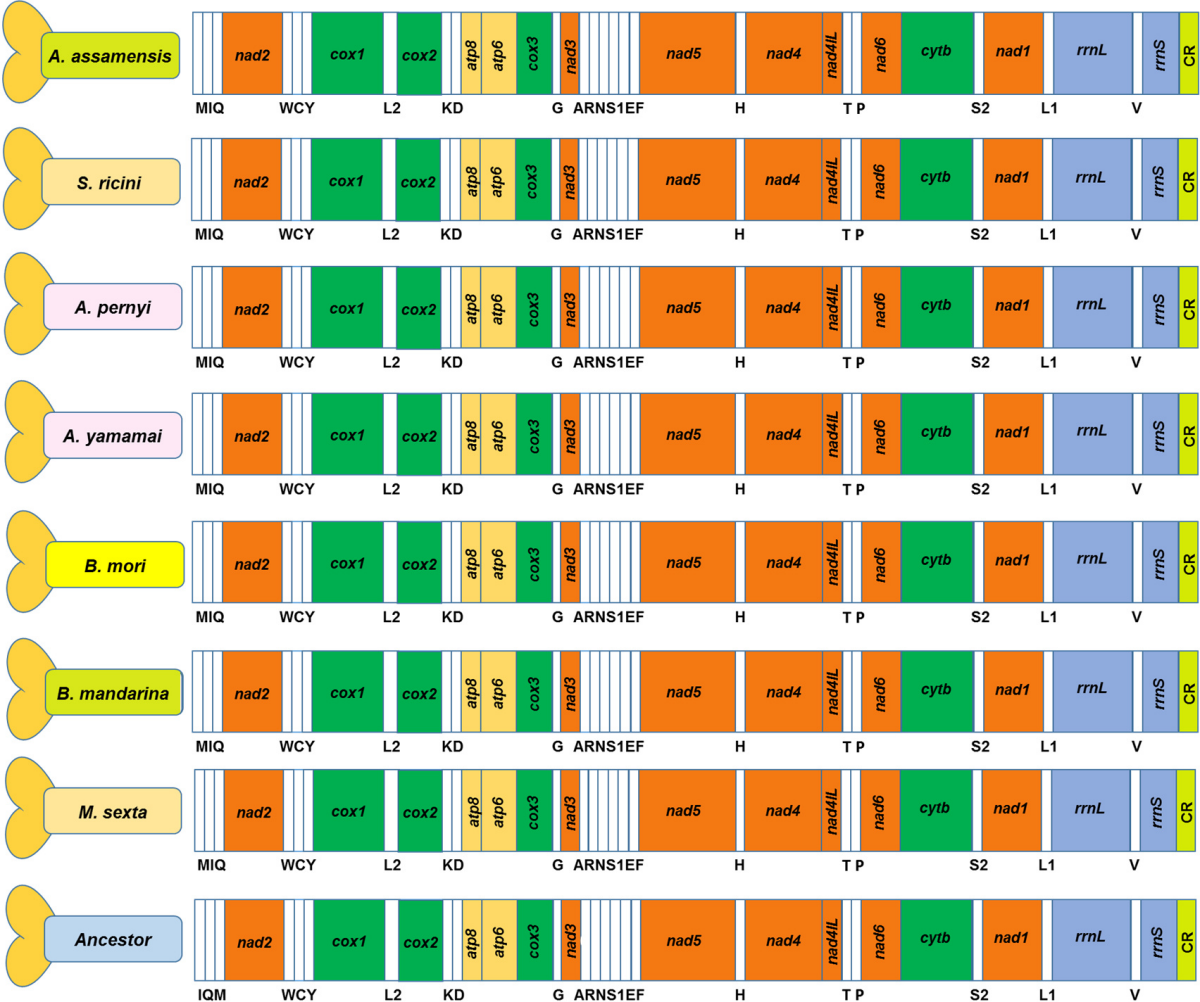
Mitogenome arrangement of *A*. *assamensis* with respect to other selected species. Ancestor here denotes *Thitarodes renzhiensis*.

The existence of a typical gene content i.e. 37 genes and a control region was evident in *A*. *assamensis* mitogenome as observed in other sequenced mitogenomes of lepidopteran insects. The arrangement of mitochondrial genes was found to be in the order identical to the selected Bombycoid insects (Fig 3). Three typical rearrangements were observed in the mitogenome of *A*. *assamensis* similar to most of the other lepidopteran insects [35, 37, 44, 45] as shown in Fig 3. These were (i) *tRNA*^*Met-Ile-Gln*^ (*trnM/trnI/trnQ* or *MIQ*) cluster, (ii) *tRNA*^*Lys-Asp*^ (*K-D*) cluster and (iii) *tRNA*^*Ala-Arg-Asn-Ser1-Glu-Phe*^ (*ARNS1EF*) cluster. The typical *MIQ* cluster rearrangement located between the control region and *nad2* gene differed from the ancestral pattern *tRNA*^*Ile-Gln-Met*^ (*trnI/trnQ/trnM* or *IQM*) as reported in two ghost moths (Fig 3) belonging to Hepialidae family—a non-ditrysian lineage (Lepidoptera: Exoporia: Hepialoidea) [63–64]. This suggests that *A*. *assamensis* along with other lepidopteran insects have evolved with a typical gene arrangement after splitting from its stem lineage during the course of time. A typical *ARNS1EF* arrangement is found in most of the lepidopteran insects. However, some rearrangement in this cluster has been reported in few lepidopteran insects different from *A*. *assamensis* [65–66]. Further, sequence similarity performed among selected Bombycoids showed that *A*. *assamensis* shared highest similarity with *Antheraea* species (*A*. *yamamai*—92.1%, *A*. *pernyi*—91.7%) followed by the other Bombycoids like *S*. *ricini* (88.9%), *M*. *sexta* (85.5%), *B*. *mori* (83.4%) and *B*. *mandarina* (82.9%) indicating a significant level of homology among Bombycoidea superfamily of Lepidoptera.

#### Comparative analysis among protein coding genes (PCGs)

The mitogenome of *A*. *assamensis* comprised of a total of 13 PCGs which spanned 11,208 bp constituting around 73.38% of the total mitogenome. The total size of PCGs and their individual sizes were found to be similar to the other Bombycoid species (S1 Table). The PCGs were distributed over both the strands of double stranded mitogenome, 9 genes in the J-strand and 4 genes in the N-strand.

The ATN sequence was found to be the start codon for all the genes in *A*. *assamensis* similar to other Bombycoid insects [24, 25, 26, 31, 33]. These codons include ATT (*nad2*, *atp8*, *nad5*), ATA (*nad3*, *nad6*, *cytb*) and ATG (*atp6*, *cox3*, *nad4*, *nad4l*, *nad1*) (Fig 2B). Interestingly, start codons of *cox1* and *cox2* genes in *A*. *assamensis* were found to deviate from the canonical ATN initiation codon pattern. Similarly, *A*. *pernyi* [25], *A*. *yamamai* [26], *S*. *boisduvalii* [28], *B*. *mandarina* [30], *B*. *mori* [31] and *M*. *sexta* [33] were also reported to diverge from this pattern. In addition to the gene sequence similarity analysis, comparative amino acid sequence alignment of the reported lepidopteran insects confirmed CGA and GTG as the initiation codons for both *cox1* and *cox2* genes of *A*. *assamensis*, respectively. CGA is also reported as the start codon for *cox1* gene in some of the members of Lepidoptera such as *A*. *atlas* [23], *E*. *pyretorum* [29], *M*. *sexta* [33], *A*. *honmai* [45], *Maruca vitrata* [67] and dipteran insect such as *Drosophila melanogaster* [68]. On the other hand, GTG is the start codon for *cox2* gene in *S*. *ricini* [24], *S*. *boisduvalii* [28] and *E*. *pyretorum* [29].

Further, three genes *cox1*, *cox2* and *nad5* showed incomplete termination codon (only T) instead of canonical termination codon pattern TAA or TAG (Fig 2B). This is similar to *A*. *pernyi* which also shows incomplete termination codon in *cox1*, *cox2* and *nad5* genes [25]. The existence of incomplete stop codon has been observed as a common phenomenon of mitochondrial genes of metazoans like lepidopterans in order to minimize IGS and OS [25, 26, 31, 33, 49]. Several studies reveal that it could be a recognition site for an endonuclease that truncates the polycistronic pre-mRNA. These incomplete codons are rectified by the post-transcriptional polyadenylation to yield functional stop codon with TAA termini [33].

The PCGs of *A*. *assamensis* were also compared with seven different organisms belonging to various levels of taxonomic classification in Insecta class at nucleotide level and corresponding amino acid level in order to determine the conserved, variable and phylogenetically informative sites (S2 Table). Various species of *Antheraea* showed 84–90% conserved sites at nucleotide level while at amino acid level it was comparatively higher (86–98%). This might be due to synonymous substitution where a non-beneficial mutation is purified by same amino acid encoding codons. However, *atp8* gene exhibited lower values (76.8%) at amino acid level which may suggest that mutation in the gene was needed to help the organism in its evolution. Various comparative analyses were further carried out for the organisms of different genus, family, superfamily and order. The conserved sites were found to decrease with higher hierarchical organisms while variable and phylogenetically informative sites increased in number. Among all PCGs, *cox1* and *cox2* genes showed maximal conservation both at nucleotide and amino acid levels across the organisms of various classification levels. Similarly, *M*. *sexta* and *P*. *maraho* were also reported to exhibit higher conservation for *cox1* and *cox2* genes [33, 40].

The study of nucleotide variation pattern (transition-transversion ratio) among the PCGs of *A*. *assamensis* with respect to various members (*A*. *pernyi*, *A*. *yamamai*, *S*. *ricini*, *M*. *sexta*, *B*. *mori* and *B*. *mandarina*) of Bombycoidea superfamily revealed that the transition-transversion ratio decreased with distant organisms in comparison with closer ones across all the PCGs [69]. For example, ratio for *atp8* gene varied from 1.7 to 0.1 when *A*. *assamensis* was compared with its close relative *A*. *pernyi* (same genus) to distant organism *M*. *sexta* (different family). However in case of *nad4l* gene, a lower ratio was observed for *A*. *pernyi* (0.24) than distant ones. Further comparative analysis of all the sequenced species of *Antheraea* genus was carried out in order to find out variation in *nad4l* gene within the genus. The *nad4l* gene of *A*. *assamensis* showed lower ratio across the species whereas higher ratio was observed in comparison with other species. Interestingly, it exhibited higher ratio with *S*. *ricini* which belongs to different genus of same family. This apparently depicts that *nad4l* gene is a decisive marker for evolution to form *A*. *assamensis* within *Antheraea* genus. Further, we may hypothesize that *A*. *assamensis* might have acquired gene from *S*. *ricini* during its evolution as both the organism are found in Northeast India and share the same evolutionary space [70].

**Fig 4:**
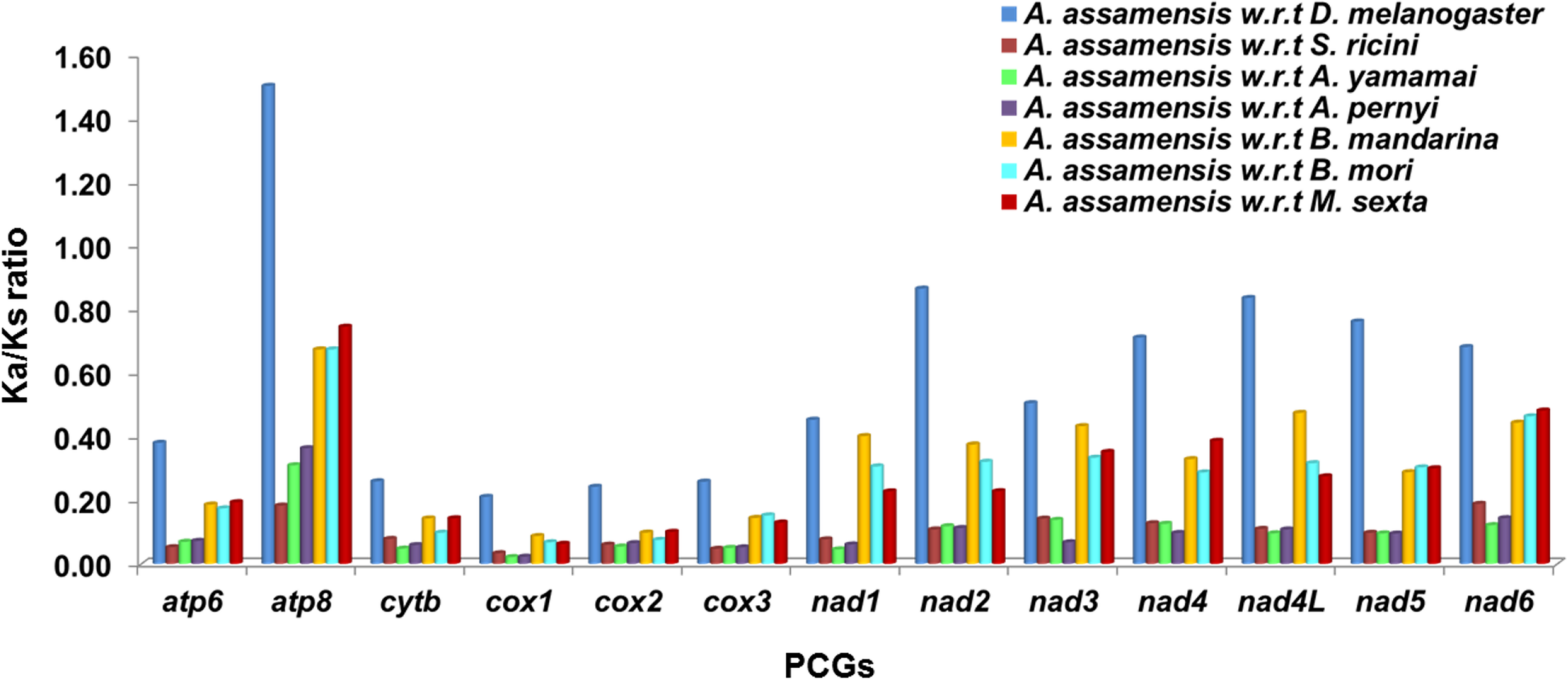
Evolutionary rates (Ka/Ks) of individual PCG of *A*. *assamensis* with selected species. w.r.t. here denotes with reference to-.

Different evolutionary rates among the genes reflect different functional constraints affecting the mitogenome that varies among species. Therefore, in order to determine the evolutionary rates in *A*. *assamensis*, the PCGs were compared with that of seven different organisms belonging to various hierarchy levels in Insecta class. The average rate of synonymous substitutions (Ks) and the average rate of non-synonymous substitutions (Ka) along with their ratios (Ka/Ks) were calculated for all 13 PCGs. The ratio Ka/Ks less than, greater than and equal to 1 indicates that genes are under negative (purifying) selection, positive (adaptative) selection and neutral evolution, respectively [71]. Among 13 PCGs, *atp8* gene encoding ATPase subunit 8 exhibited 1.5 ratio with reference to that of *D*. *melanogaster* indicating positive/ relaxed selection acting on this gene (Fig 4). This indicates that mutation in *atp8* of *A*. *assamensis* was due to a necessity to re-organize its structure. These changes may be due to the requirement of energy for the production and secretion of silk fibers which are not observed in *D*. *melanogaster*. It depicts that functional change in an organism needs commitment mutation in responsible genes in order to support survival of the fittest. Notably, the ratio for remaining PCGs of *A*. *assamensis* with all organisms was found to be less than one which suggests that mutation was against the requirement and hence mutation was replaced by synonymous nucleotides. This suggests that all the protein coding genes evolved under strong purifying (negative) selection as is evident from the number of conserved and variable sites. The Ka/Ks ratio in *atp8* with reference to *B*. *mandarina*, *B*. *mori* and *M*. *sexta* was found to be significantly higher. These organisms belong to different family with respect to *A*. *assamensis* and hence significant variation in *atp8* gene can be expected due to certain changes in the mitogenome kinetics. As *S*. *ricini*, *A*. *yamamai*, *A*. *pernyi* belong to the same family of *A*. *assamensis*, significant Ka/Ks ratio was not observed across the PCGs. Overall comparison of PCGs exhibited that mutation in *atp8* gene was essential in insects to yield several strains with different kinetics as other PCGs showed lower ratios. The lowest evolutionary rates were observed for *cox1* gene indicating that it is least susceptible to variation in protein sequence and hence shows potential as a barcode for evolutionary studies in silkworms. In addition, other genes like *cox2*, *cox3*, *cytb* and *nad* genes with slightly low evolutionary rates next to *cox1* can also serve as barcode markers. Furthermore, the effect of GC content on Ka/Ks ratio was studied and we found a significant negative correlation between them (S2 Fig). This indicates that change in GC content may result in the variation in nucleotide substitution pattern or evolutionary pattern among the PCGs.

#### Comparative analysis among transfer RNAs (tRNAs)

Twenty two tRNA genes with a total length of 1465 bp were found to be present in the mitogenome of *A*. *assamensis*. 14 tRNA genes were encoded by the J strand while 8 tRNA genes were encoded by the N-strand (Fig 2B). The individual length of each gene varied from 64 to 71 bp as reported for other lepidopterans (S1 Table).

The secondary structures of tRNAs of *A*. *assamensis* exhibited a typical clover-leaf structure similar to other lepidopteran species (S3 and S4 Figs). This depicts that arrangement of all the tRNA genes is mostly conserved among insects. However, some variation in the structures of *A*. *assamensis* was observed such as aminoacyl acceptor stem of *tRNA*^*Met*^, TΨC loop of *tRNA*^*Ile*^, *tRNA*^*Cys*^, *tRNA*^*Tyr*^, *tRNA*^*Arg*^, *tRNA*^*Asn*^, *tRNA*^*Glu*^, *tRNA*^*Phe*^, *tRNA*^*Trp*^, *tRNA*^*Ser2*^, dihydrouridine (DHU) arm of *tRNA*^*Ser1*^, etc. The lack of stable DHU arm in *tRNA*^*Ser1*^ and TΨC loop in *tRNA*^*Tyr*^ has also been reported in lepidopterans such as *S*. *ricini*, *A*. *yamamai*, *A*. *pernyi*, *B*. *mori*, *B*. *mandarina*, *M*. *sexta*, *E*. *pyretorum*, *A*. *fylloides*, *B*. *panterinaria*, *E*. *autonoe*, etc. [24, 25, 26, 29, 30, 31, 33, 35, 38, 42]. These structural variations might be attributed to variation in the number of base pairs responsible for the formation of aminoacyl acceptor (AA) stem, DHU arm, TΨC arm and anticodon (AC) stem-loop (S5 and S6 Figs). Among various structural parts of tRNAs, AA stem of *A*. *assamensis* was found to be more consistent in size across all the tRNAs and in other lepidopterans as observed from comparative analysis. AC stem, AC loop and DHU stem also showed consistency in the number of base pairs across lepidopterans. However, the consistency of these stems and loops varied with the type of tRNAs. In addition, anticodons of tRNAs were found to be identical to the selected Bombycoids (S3 Table) and majority of other lepidopteran insects [26, 28, 30, 35]. On the contrary, TѰC stem, TѰC loop and DHU loop exhibited randomness in the number of base pairs across the organisms and tRNAs. For instance, TѰC loop was absent in *tRNA*^*Tyr*^ of *A*. *assamensis* while *tRNA*^*Trp*^ of *A*. *assamensis* and *tRNA*^*Tyr*^ of *B*. *mori* had 7 bp and 5 bp loops, respectively. The consistency in the number of base pairs of AA stem, AC stem, AC loop and DHU stem was probably to avoid dysfunctioning of tRNAs actively participating in protein synthesis and thus critical for the survival in the evolutionary milieu.

We identified a total of 35 mismatched base pairs in tRNAs of *A*. *assamensis* scattered over the AA stem, TΨC stem, AC and DHU regions of 19 tRNAs. 28 mismatches were observed in G-U combinations while 6 and 1 mismatches were found to be in U-U and G-A combinations, respectively (S3 Table). These mismatches lead to changes in tRNA structures; for instance, two U-U mismatches in *tRNA*^*Ser2*^ resulted in the formation of an extra loop in AC stem.

From the comparative analysis, we found that the number of total mismatches was higher in *A*. *assamensis* (35 No.s) than the selected Bombycoids like *S*. *ricini* (32 No.s), *B*. *mori* (29 No.s), *M*. *sexta* (25 No.s), etc. Numerically, this mismatch was higher than many Saturniids like *E*. *pyretorum* (24 No.s), *S*. *cynthia* (28 No.s), etc. Similarly, mismatches such as G-U, U-U, G-A, A-A, A-C and C-U have been previously reported for many lepidopterans like *E*. *autonoe* (26 No.s), *A*. *fylloides* (27 No.s), etc. [38, 42]. Among various mismatches in *A*. *assamensis*, G-U mismatches were found be the highest (28 No.s) than the selected Bombycoids and other lepidopterans e.g. *A*. *atlas* (25 No.s) and *E*. *autonoe* (20 No.s). The details of mismatches found in various stem-loop structures of different tRNAs of Bombycoid members are compared in S3 Table. The non-canonical pairing might occur due to the insertion/deletion of nucleotides or nucleotide substitution of bases within the tRNA genes as a form of ancient insertional editing. The presence of high number of mismatches indicates higher nucleotide substitution in the tRNA sequences of *A*. *assamensis* as compared to the selected Bombycoids which may result in genome structure and sequence evolution. These mismatches are likely to be corrected by RNA editing mechanism as observed in other arthropods [72].

#### Comparative analysis among ribosomal RNAs (rRNAs)

*A*. *assamensis* comprised of two genes for rRNA coded by (–) strand of the mitogenome. *rrnL* (*lrRNA*) is located between *tRNA*^*Leu1*^ and *tRNA*^*Val*^ genes, whereas *rrnS* (*srRNA*) is accommodated between *tRNA*^*Val*^ and control region as reported for other lepidopterans. The lengths of *rrnL* and *rrnS* were 1344 bp and 779 bp, respectively (S1 Table) and thus similar to many reported lepidopteran insects [38, 43]. The sequence homology of two rRNA genes of *A*. *assamensis* with the selected Bombycoids revealed that the sequence identity of *rrnS* gene (84 to 95%) was higher than that of *rrnL* (80 to 89%). The divergence study in rRNA genes (*rrnL* and *rrnS*) also exhibited low variability sites which suggest that these are highly conserved sites and may have potential application in molecular systematics. This may be due to the natural effort to preserve the structure and function of rRNA as protein synthesis depends on it.

#### Comparative analysis among control region (A+T rich region)

**Fig 5:**
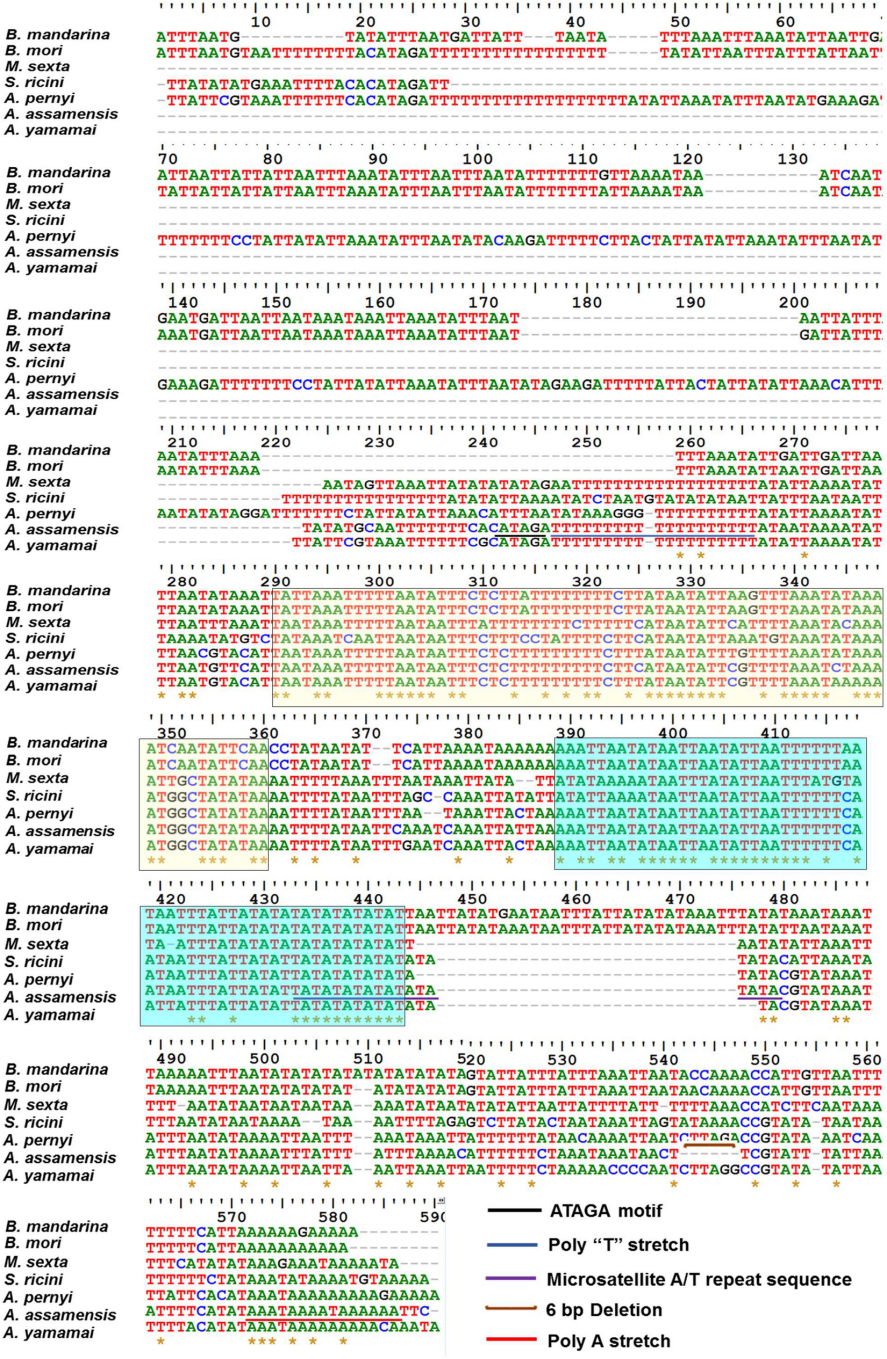
Multiple sequence alignment of control region of *A*. *assamensis* mitogenome showing conserved structural elements. Yellow and green highlighted blocks represent 1^st^ and 2^nd^ conserved blocks, respectively.

The control region is the longest non-coding region of *A*. *assamensis* mitogenome and with a length of 328 bp, is similar to other Bombycoids. This region is located between *rrnS* and *MIQ* cluster. The comparative sequence homology of control region displayed higher similarity with its close relatives such as *A*. *yamamai* (89.2%) and *A*. *pernyi* (82.7%) while showed lower similarity with distant organisms such as *B*. *mori* (~68%). It was found that two sequence blocks of more than 50 bp in length displayed 96–100% similarity with other *Antheraea* species while *S*. *ricini* (78–96%), *M*. *sexta* (68–88%) and *B*. *mori* (73–80%) showed low similarity. Additionally, some conserved structures and repeats were observed in the control region of *A*. *assamensis* (Fig 5). These were ‘ATAGA’ motif, poly T stretch (T)_19_, microsatellite (TA)_9_ repeat sequence, poly-A region and a 6 bp deletion. ‘ATAGA’ motif and (T)_19_ are known to be important for gene regulation and serve as a recognition site for replication initiation of minor or light strand. The poly A tail has been proposed to be required for RNA maturation and serve as a sequence for controlling transcription or replication initiation in insects [73]. The microsatellites (TA)_9_ also known as simple sequence repeats (SSR) are proposed to be used as molecular markers due to their abundance and highly polymorphic nature [24]. A microsatellite (AT)n element is a well conserved site and can be used for evolutionary and conservation genetic studies [25, 26, 74]. The control region of *A*. *assamensis* lacked tandem repeat elements similar to other completely sequenced *Antheraea* species except *A*. *pernyi* which harbored six 38 bp tandem repeats. The significance of these tandem repeats is unclear and needs further taxon sampling and mitogenome characterization. Presence of highly conserved yet distinct consensus sequences in the control region thus shows its high potential as a phylogenetic marker at lower taxonomic levels.

#### Comparative analysis among overlapping sequence (OS) and intergenic spacer (IGS) regions

The overlapping sequences (OS) and intergenic spacers (IGS) are commonly found in the mitogenome of lepidopterans. These regions may vary in length and location from species to species during their evolution. In case of *A*. *assamensis*, six OS and seventeen IGS were found to be present in the mitogenome. The size of OS and IGS were in the range of 1–8 bp (cumulative length of 23 bp) and 1–50 bp (cumulative length of 171 bp), respectively. The presence of OS, IGS and their corresponding lengths were similar to many of the reported lepidopterans [23, 27, 32, 43, 45] as given in S4 Table. A characteristic conserved overlapping junction (ATGATAA) was observed in between *atp8* and *atp6* genes as reported for most of the lepidopterans [38, 42, 45].

The spacer between the genes *tRNA*^*Gln*^ and *nad2* (*tRNA*^*Gln*^*—nad2* spacer) was found to be the largest (50 bp) in *A*. *assamensis* but slightly shorter than that observed in *A*. *yamamai* (53 bp), *S*. *cynthia* (54 bp), etc. Similarly, it has been detected as the largest spacer in many other lepidopterans like *A*. *atlas*, *A*. *selene*, *S*. *boisduvali*, *E*. *pyretorum*, *P*. *bremeri* and *A*. *honmai* [23, 27, 28, 29, 41, 45]. Further, the spacers *cytb-tRNA*^*Ser2*^ (31 bp), *tRNA*^*Ser2*^*-nad1* (23 bp), *tRNA*^*Lys*^*- tRNA*^*Asp*^ (17 bp), etc. were also found to exist in *A*. *assamensis* mitogenome (Fig 2B). The spacer *cytb-tRNA*^*Ser2*^ may serve as a binding site for the mtTERM, which is a transcription termination peptide [24, 26, 33]. A conserved motif ‘ATACTAA’ was found in the spacer *tRNA*^*Ser2*^*-nad1* on the comparison of *A*. *assamensis* with selected Bombycoid species. This spacer is implicated in mitochondrial transcription termination where the ‘ATACTAA’ motif is essential as a recognition site for mtTERM [24, 26, 33].

The spacers in *A*. *assamensis* mitogenome also exhibited sequence homology with their adjacent genes as observed in many of the lepidopteran insects [24–26]. For instance, spacer *tRNA*^*Gln*^-*nad2* showed 68% similarity with its adjacent *nad2* gene. This might be because of the partial duplication of *nad2* gene in order to provide another origin of replication [26, 28, 33]. This also suggests that *A*. *assamensis* may have undergone rapid sequence divergence attributed to the non-coding nature of IGS as seen in other organisms [26]. Apart from adjacent genes, IGS also showed sequence homology with their distant genes in *A*. *assamensis*. For example, spacer *tRNA*^*Lys*^-*tRNA*^*Asp*^ showed homology with *rrnS* (82%), *nad5* (76%) and *atp8* (82%) genes. Similarly, the same spacer in *S*. *ricini* has also been reported to exhibit homology with *rrnS* (78%) and *nad5* (72%) genes [24]. This might be attributed to gene duplication and degeneration.

#### Comparative analysis with respect to nucleotide composition, skewness and codon usage

The nucleotide composition in mitogenome of *A*. *assamensis* was analyzed in terms of specific nucleotide content, AT and GC skewness. Whole mitogenome nucleotide composition revealed 80.2% AT content (40.8% T + 39.4% A) and remaining 19.8% GC content (12.1% C + 7.7% G). Similar to *A*. *assamensis*, many lepidopterans insects also showed enhanced AT content [37, 39]. This AT content was found to vary with different regions of the mitogenome. The AT content was maximal (~91.2%) in the control region followed by rRNAs genes (84.3%), tRNAs genes (80.8%) and PCGs (78%). The individual PCGs also exhibited variation in the corresponding AT content. For instance, *atp8*, *nad4l* and *nad6* genes had the highest AT content while *cox1* and *cox3* genes had lowest values. This variation pattern was also detected in the other lepidopterans like *E*. *pyretorum*, *P*. *atrilineata*, *G*. *menyuanensis*, etc. [29, 36, 43]. A close analysis of the third codon position in all 13 PCGs of *A*. *assamensis* exhibited AT biasness which was 93% than the first (73%) and second (70%) codon positions similar to the other Bombycoids [24, 25, 29]. This may be attributed to more relaxed constraints on A+T content at 3^rd^ position than at 1^st^ and 2^nd^ position due to the phenomena of degeneracy in genetic code.

Nucleotide skewness was determined to measure the relative number of Gs to Cs and As to Ts. The AT skewness in *A*. *assamensis* mitogenome was found to be negative (-0.02) which indicates the occurrence of more Ts than As and lies within the range of Saturniid moths [25–26]. This value differed from the selected Bombycidae species having positive AT skew values e.g. 0.06 in *B*. *mori*, *B*. *mandarina*, etc. [30–31]. Similarly, mitogenome GC skewness also exhibited negative value (-0.22) as observed in many Bombycoids such as *M*. *sexta* (-0.18) and *S*. *ricini* (-0.23). The GC skewness varied with major and minor strands of PCGs, however, no significant change was observed in AT skewness. Minor strand was G-skewed i.e. rich in G nucleotides while more ‘C’s were encoded by major strand (S5 Table). The strand asymmetry is common in insects and has been reported in all the lepidopterans which occur due to asymmetrical mutation pressure on mitogenome. These skewness patterns were also observed in rRNAs, tRNAs, control region and are similar to the other sequenced lepidopteran insects.

**Fig 6:**
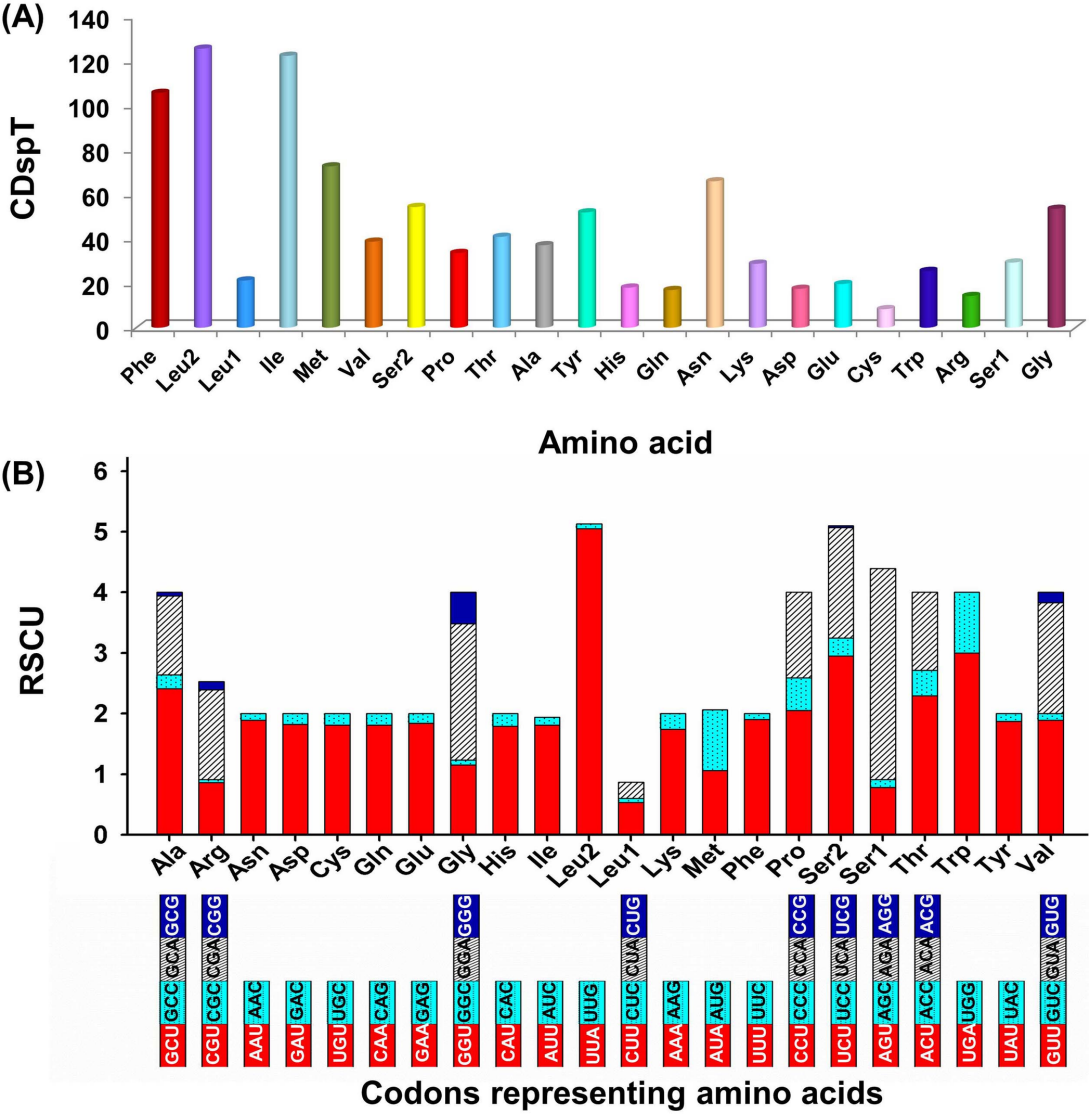
Codon usage in *A*. *assamensis* mitogenome. **(A) Codon distribution where CDspT = codons per thousand codons (B) Relative synonymous codon usage (RSCU).** Termination codons were excluded in the study.

Nucleotide biasness was also reflected in the codon usage of PCGs that determines the frequency of synonymous codon usage (SCU) in various genomes at inter and intra levels. Based on the codon usage and relative SCU results, codons with A or T nucleotides at the 3^rd^ codon position were highly used in comparison with G or C nucleotides as discussed above. The five most frequently used amino acids were Leu, Ile, Phe, Met and Asn (Fig 6) and the most prevalent codons for these corresponding amino acids were UUA, AUU, UUU, AUA and AAU with a usage frequency of 45.53% to 48.95% (S2 Fig). Cys was found to be the least used amino acid as detected in other selected Bombycoids. Biasness in SCU has been observed in many other lepidopterans to avoid errors due to mis-incorporation of amino acids which depends upon various factors like natural selection (gene length and function), mutation bias (base mutation and GC content), etc. [24, 35]. This A+T biasness may result in the change in amino acid composition similar to the other lepidopterans. Therefore, the codon usage analysis will have great significance in studying gene expression and evolutionary studies in silkworms.

#### Comparative phylogenetic analysis

**Fig 7:**
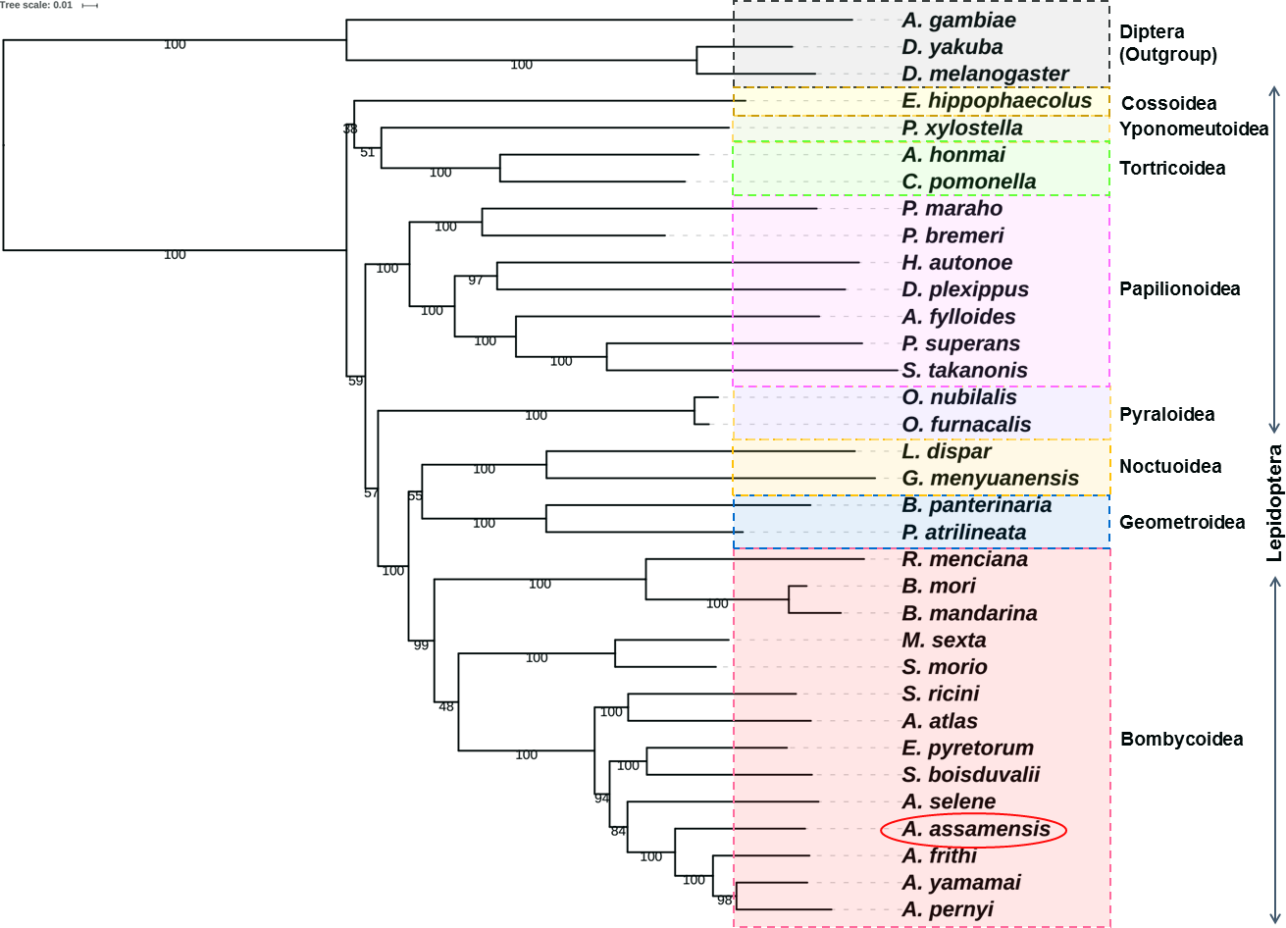
Phylogenetic tree inferred from nucleotide sequences of 13 PCGs of 34 organisms using maximum likelihood (ML) method in RaxmlGUI v1.3 (1000 bootstrap replicates). The tree is drawn to scale with bootstrap values indicated along with the branches.

**Fig 8:**
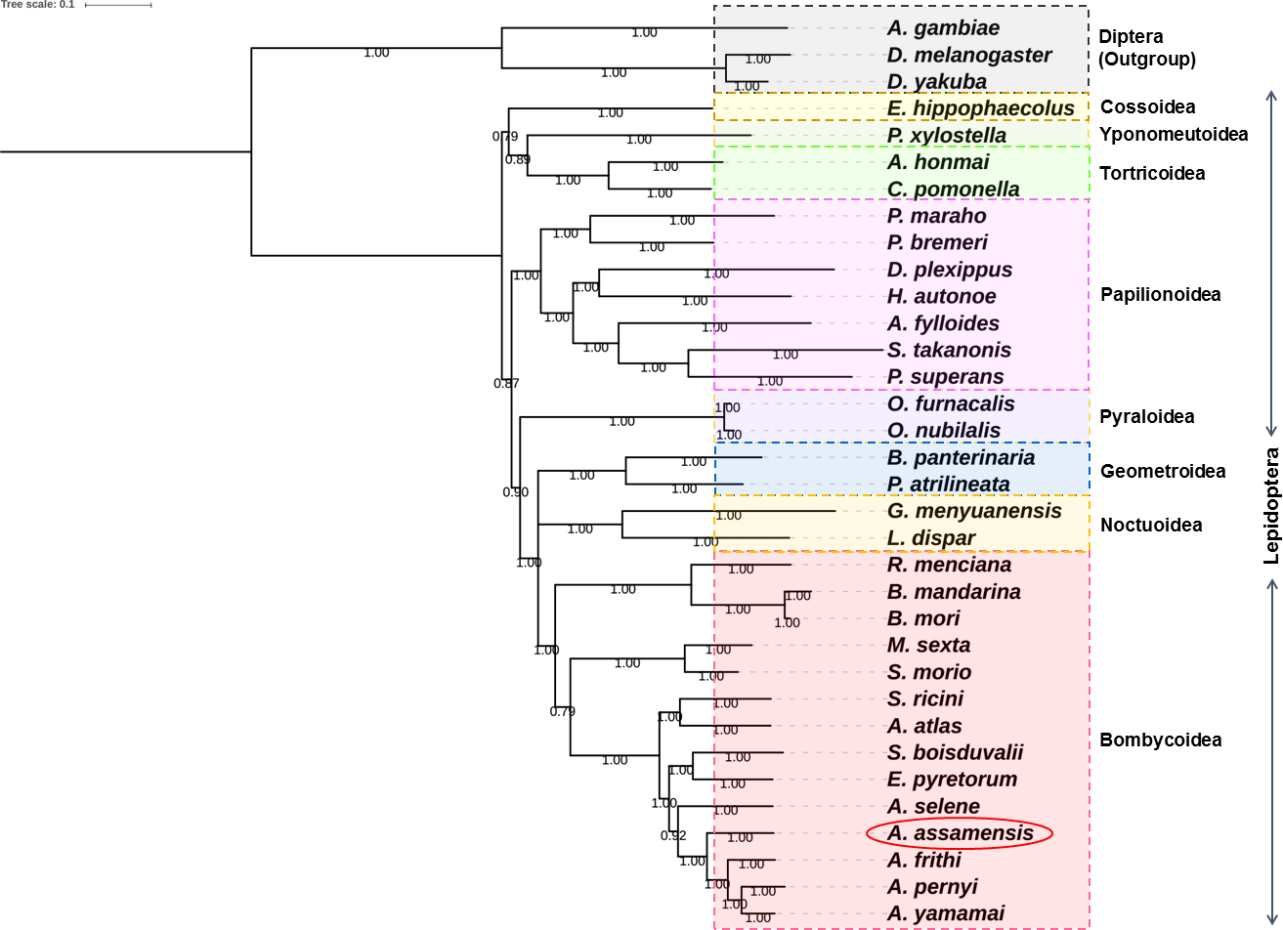
Phylogenetic tree inferred from nucleotide sequences of 13 PCGs of 34 organisms using bayesian inference (BI) method in MrBayes v3.2.6. The tree is drawn to scale with bayesian posterior probability values indicated along with the branches.

Maximum likelihood (ML) and Bayesian Inference (BI) phylogenetic trees were constructed based on nucleotide sequences of 13 PCGs and 13 PCGs+2 rRNAs datasets, respectively (Figs 7 and 8, S7 and S8 Figs). The phylogenetic trees obtained using both the methods were found to be of similar topologies. The tree clustering showed that *A*. *assamensis* belongs to Saturniidae family which is clustered in the same branch with other families (Bombycidae+Sphingidae) of Bombycoidea superfamily within the order Lepidoptera. Within the Saturniidae family, *A*. *assamensis* has clustered around with other *Antheraea* species and was also close to *A*. *selene*. Further, our study supported the triplet superfamily (Bombycoidea+Geometroidea+Noctuoidea) relationship under Macroheterocera which along with Pyraloidea and Papilionoidea formed Obtectomera, a group within Apoditrysia [8–9]. These results were consistent with the previously reported studies [26, 32, 35, 75]. More mitogenome sequence information on Bombycoidea superfamily is needed to gain deep insights into the families/superfamilies relationships within Lepidoptera. The present study supports the fact that intra and inter family level relationships are well resolved within the superfamily Bombycoidea providing a better evolutionary relationship among the silk producing insects.

## Conclusion

Our study deciphered the complete mitogenome of *A*. *assamensis* which shares similarity with majority of the lepidopterans, particularly Saturniids, in several characteristics such as genome organization and content, PCG size and structure, AT/GC skewness, tRNA structure and anti-codons, OS and IGS regions. Our analysis indicates that *A*. *assamensis* PCGs evolved under strong purifying selection and tRNAs genes showed high base substitution/mismatches. Transition-transversion ratio suggests that *nad4l* gene may be the significant contributor for evolution to form *A*. *assamensis* within *Antheraea* genus. A six-bp indel (deletion) and two highly conserved sequence blocks in control region suggest its potential application as a marker for delineating other closely related *Antheraea sp*. and subsequent usage in phylogenetic studies at lower taxonomic levels. The largest *tRNA*^*Gln*^*-nad2* spacer found in the *A*. *assamensis* mitogenome may serve as another origin of replication. Our study assigns the taxonomic status of *A*. *assamensis* using an optimal model based phylogenetic tree construction. This is the 12^th^ representative organism of Saturniidae family with a completely sequenced mitogenome. As *A*. *assamensis* is the sole producer of unique golden Muga silk which is the backbone of Assam’s sericulture industry, our study on its mitogenomic landscape is an important addition to the existing genome informatics resources on silkworms.

## Supporting information

S1 Fig Bioanalyzer profiles after sonication of enriched mitochondrial DNA sample and libraries. (TIF)

S2 Fig (A) G+C content versus Ka/Ks ratios of 13 concatenated PCGs in *A*. *assamensis* mitogenome (B) The usage rate of five frequently used codons in Bombycoidea members.
(TIF)

S3 Fig Secondary structures of tRNAs of *A*. *assamensis* from *trnM* to *trnR*. *trnY* lacked TΨC loop. *tRNA* here are represented as *trn*. AA denotes amino acyl arm and AC denotes anticodon arm.
(TIF)

S4 Fig Secondary structures of tRNAs of *A*. *assamensis* from *trnN* to *trnV*. *trnS1* lacked DHU loop. *tRNA* here are represented as *trn*. AA denotes amino acyl arm and AC denotes anticodon arm.
(TIF)

S5 Fig Variation in the secondary structures of *trnM*, *trnI*, *trnC*, *trnY*, *trnR* and *trnN* in *A*. *assamensis* and selected Bombycoids. AA denotes Aminoacyl arm and AC denotes Anticodon arm. S-L denotes Stem-Loop of tRNA gene. tRNA is represented as trn.
(TIF)

S6 Fig Variation in the secondary structures of *trnS1*, *trnE*, *trnF* and *trnT* in *A*. *assamensis* and selected Bombycoids. AA denotes Aminoacyl arm and AC denotes Anticodon arm. S-L denotes Stem-Loop of tRNA gene. tRNA is represented as trn.
(TIF)

S7 Fig Phylogenetic tree inferred from 13PCGs+2rRNAs of 34 organisms using maximum likelihood (ML) method in RaxmlGUI v1.3 (1000 bootstrap replicates). The tree is drawn to scale with bootstrap values indicated along with the branches.
(TIF)

S8 Fig Phylogenetic tree inferred from 13PCGs+2rRNAs of 34 organisms using Bayesian inference (BI) method in MrBayes v3.2.6. The tree is drawn to scale with bayesian posterior probability values indicated along with the branches.
(TIF)

S1 Table Comparative gene lengths in Bombycoids. (PDF)

S2 Table Gene by gene divergences in the protein coding genes at different taxonomic levels, both at nucleotide level and amino acid levels. C here denotes conserved sites, I denotes phylogenetically informative sites, V denotes variable sites, N denotes nucleotide level, A denotes amino acid level. The number of sites is represented in terms of percentage values.
(PDF)

S3 Table Anticodons (AC) of tRNAs and number of mismatches present in Amino-acyl (AA) stem, TΨC-stem, DHU-stem and Anticodon (AC) stem of mitochondrial tRNAs of *A*. *assamensis* with respect to the selected Bombycoids. (PDF)

S4 Table Intergenic spacers (IGS) and overlapping sequence (OS) between different genes in *A*. *assamensis* with respect to the selected Bombycoid species. (PDF)

S5 Table AT skewness and GC skewness in the whole mitogenome sequence, protein coding genes (PCGs), tRNAs, rRNAs and control region of *A*. *assamensis* and the selected Bombycoid species. (PDF)
