# Supplementary figures and images for: The mitochondrial genome of Muga silkworm (Antheraea assamensis) and its comparative analysis with other lepidopteran insects
Source: PLoS One. 2017 Nov 15;12(11):e0188077. doi: 10.1371/journal.pone.0188077 (PMC5687760; doi:10.1371/journal.pone.0188077)

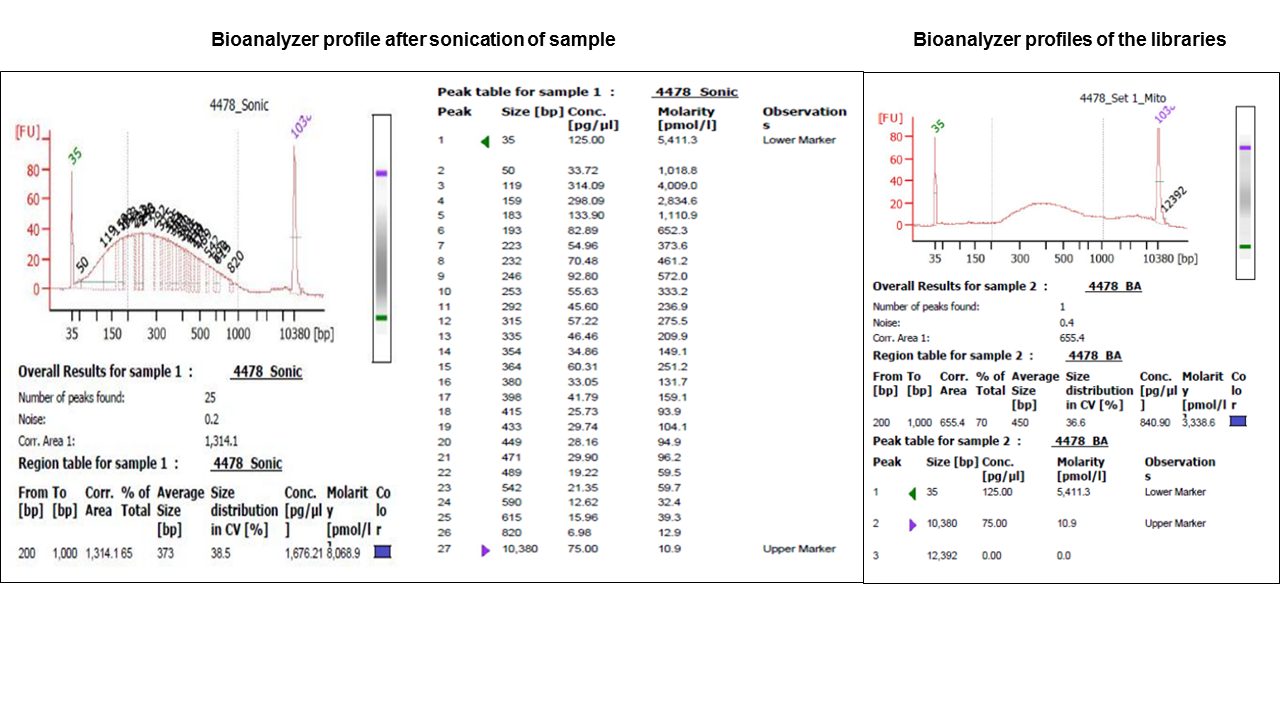

Supplement: S1 Fig — (TIF) [file pone.0188077.s001.tif]

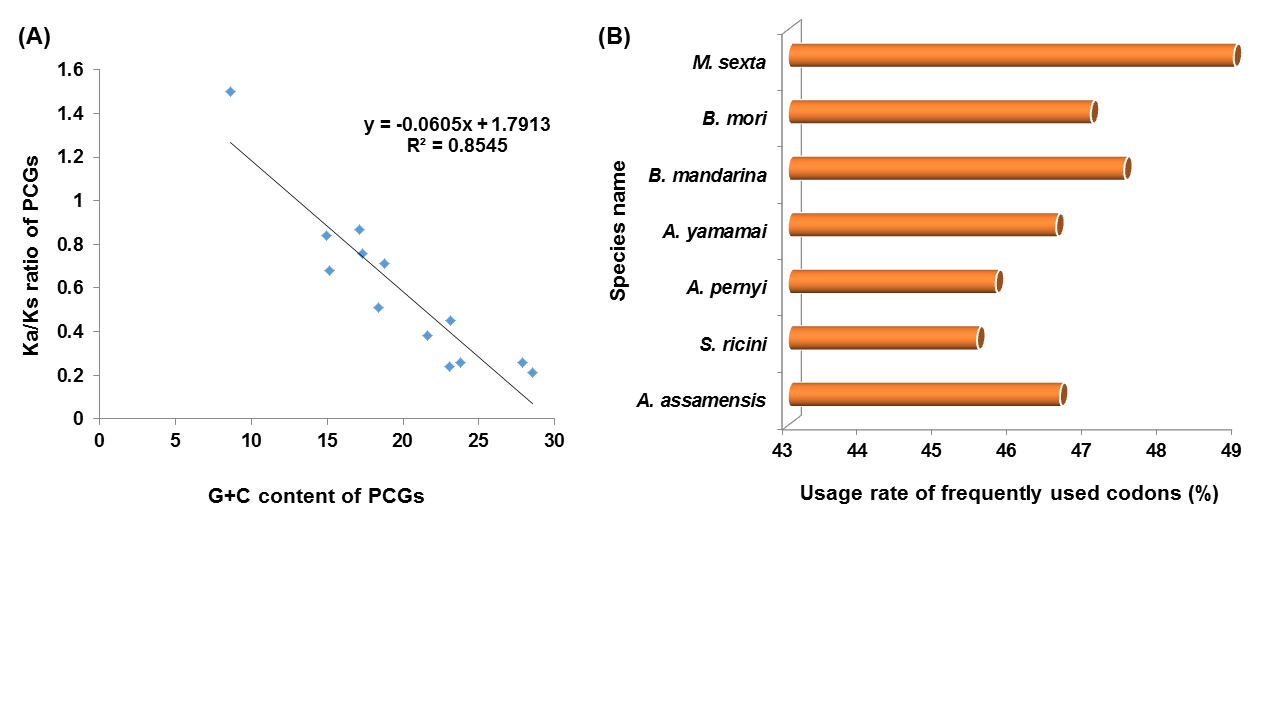

Supplement: S2 Fig — (A) G+C content versus Ka/Ks ratios of 13 concatenated PCGs in A. assamensis mitogenome (B) The usage rate of five frequently used codons in Bombycoidea members. (TIF) [file pone.0188077.s002.tif]

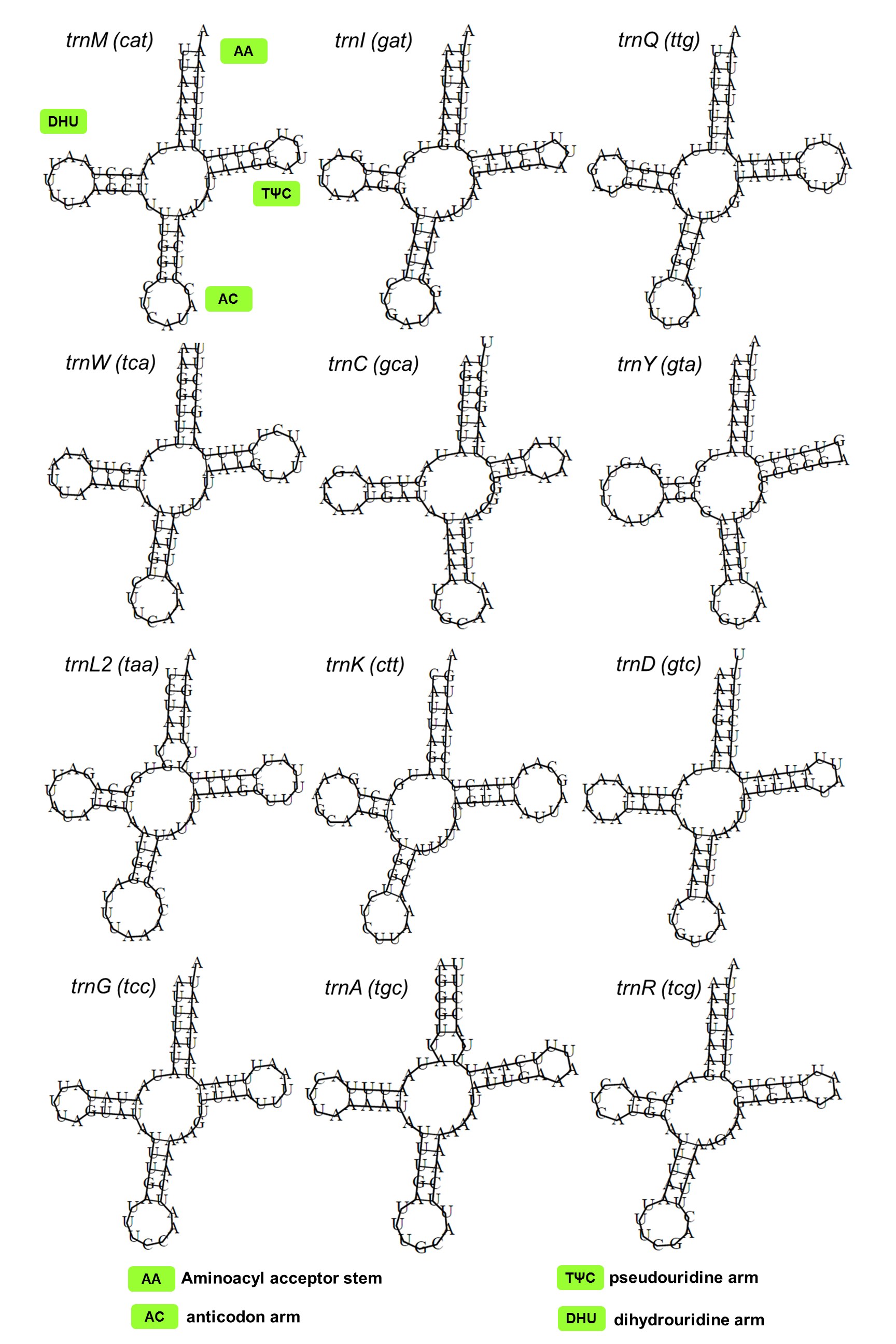

Supplement: S3 Fig — trnY lacked TΨC loop. tRNA here are represented as trn. AA denotes amino acyl arm and AC denotes anticodon arm. (TIF) [file pone.0188077.s003.tif]

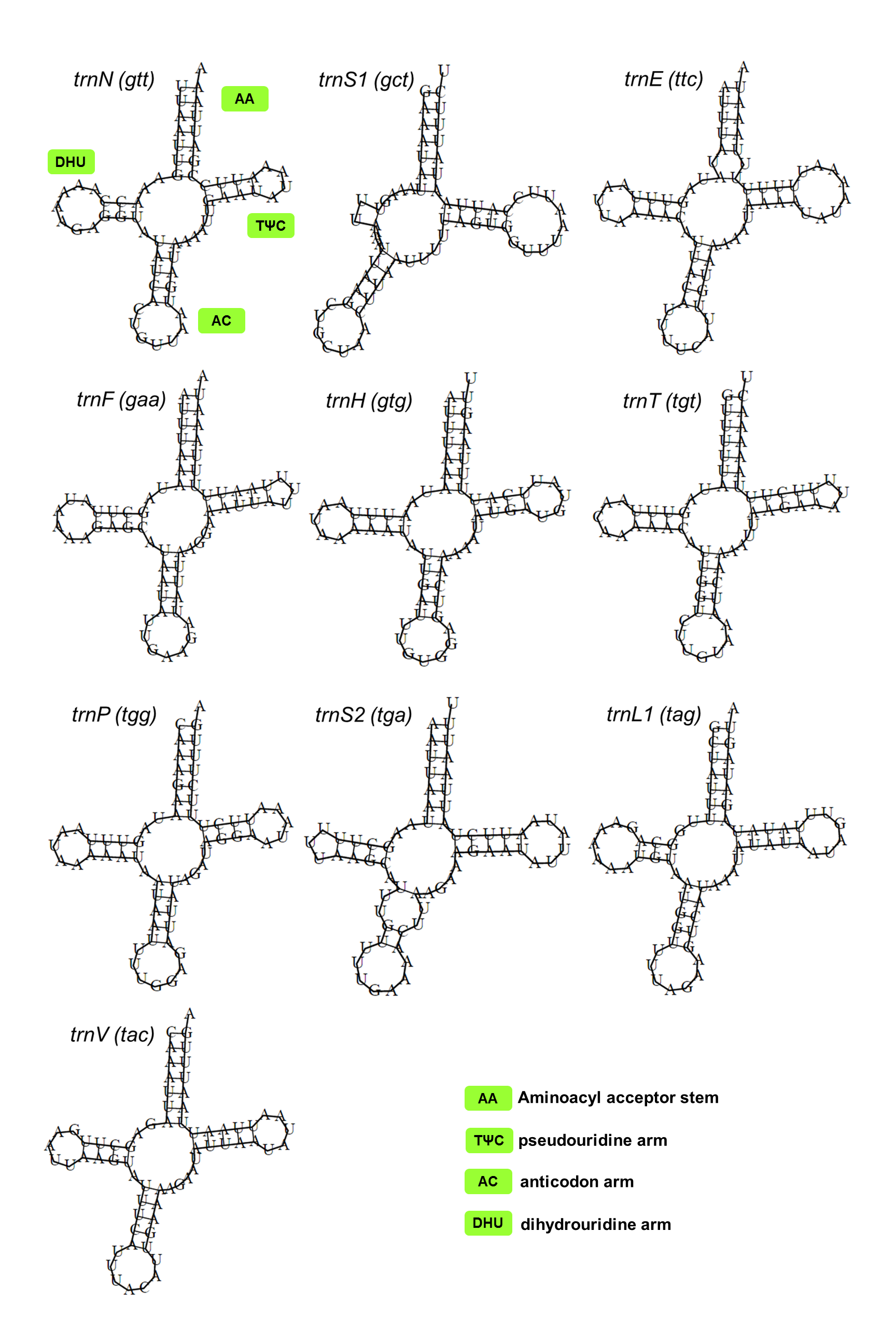

Supplement: S4 Fig — trnS1 lacked DHU loop. tRNA here are represented as trn. AA denotes amino acyl arm and AC denotes anticodon arm. (TIF) [file pone.0188077.s004.tif]

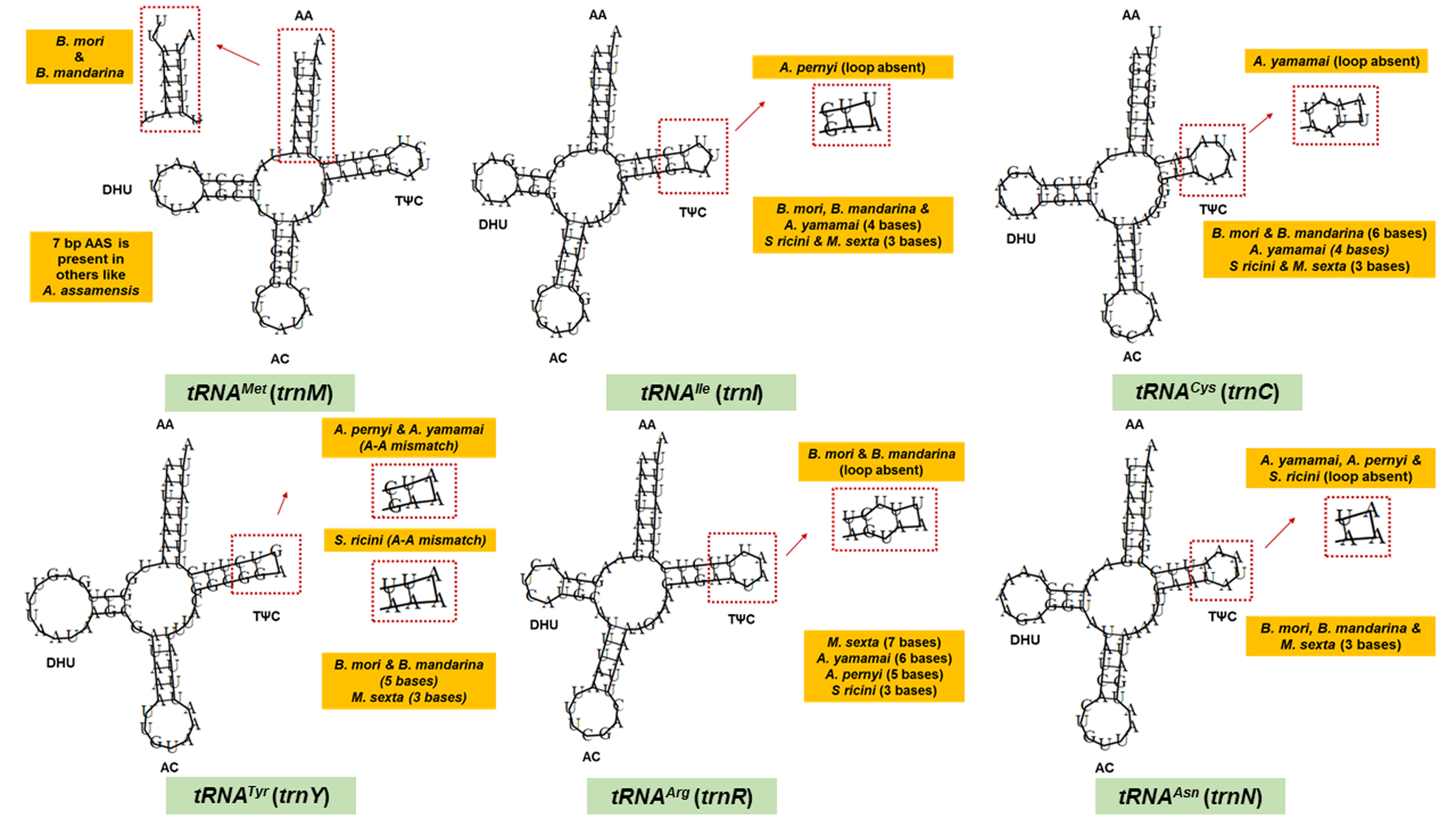

Supplement: S5 Fig — AA denotes Aminoacyl arm and AC denotes Anticodon arm. S-L denotes Stem-Loop of tRNA gene. tRNA is represented as trn. (TIF) [file pone.0188077.s005.tif]

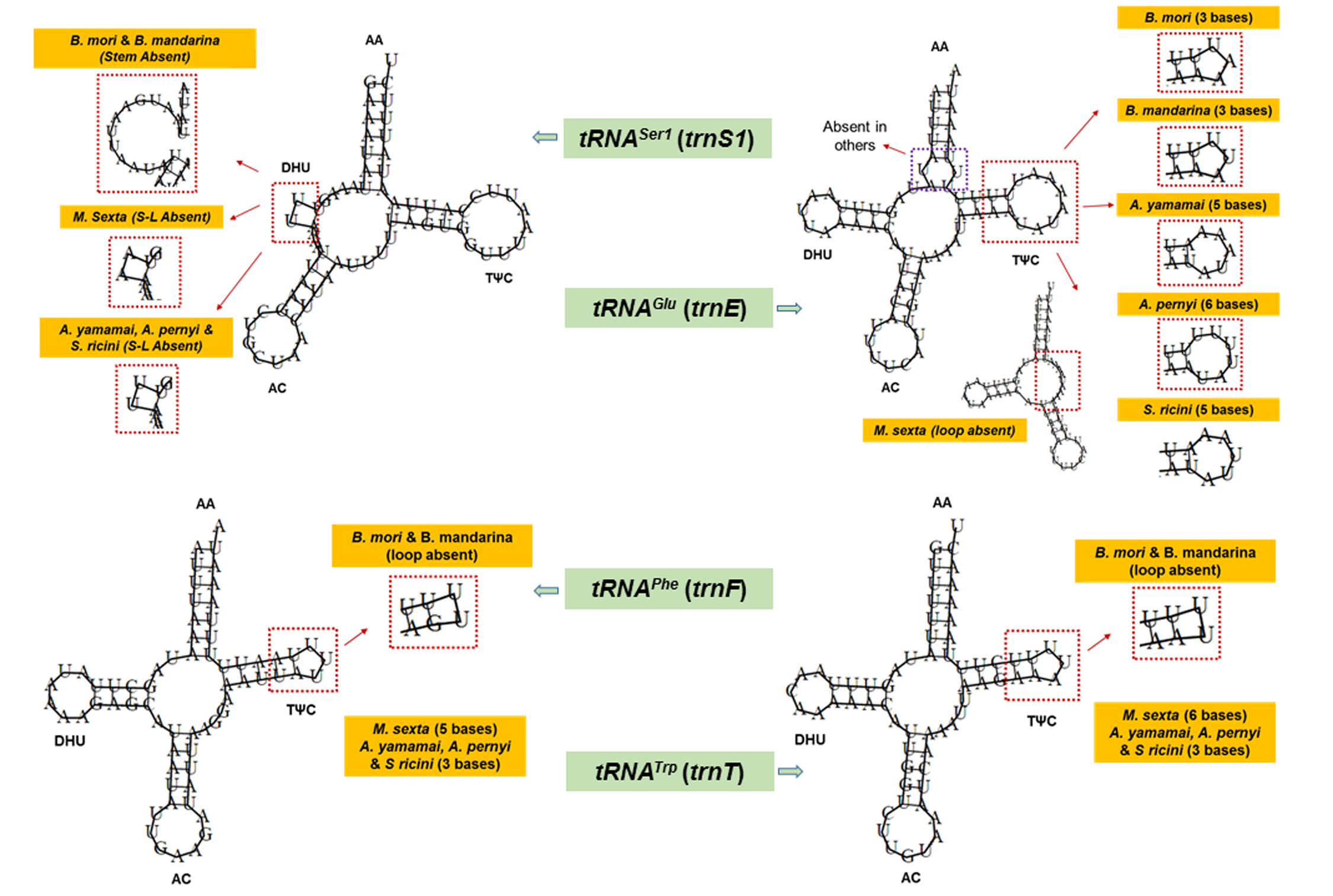

Supplement: S6 Fig — AA denotes Aminoacyl arm and AC denotes Anticodon arm. S-L denotes Stem-Loop of tRNA gene. tRNA is represented as trn. (TIF) [file pone.0188077.s006.tif]

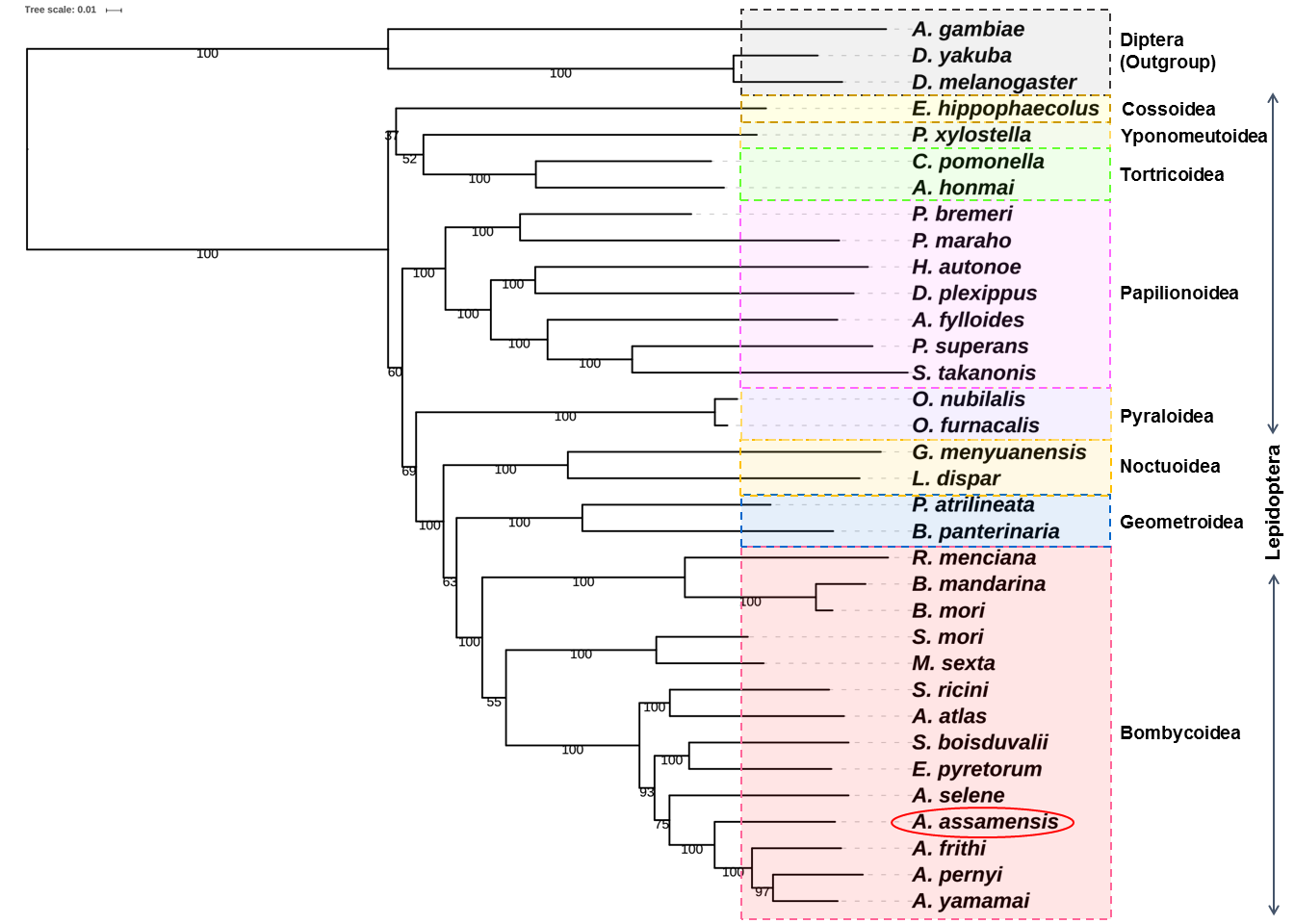

Supplement: S7 Fig — The tree is drawn to scale with bootstrap values indicated along with the branches. (TIF) [file pone.0188077.s007.tif]

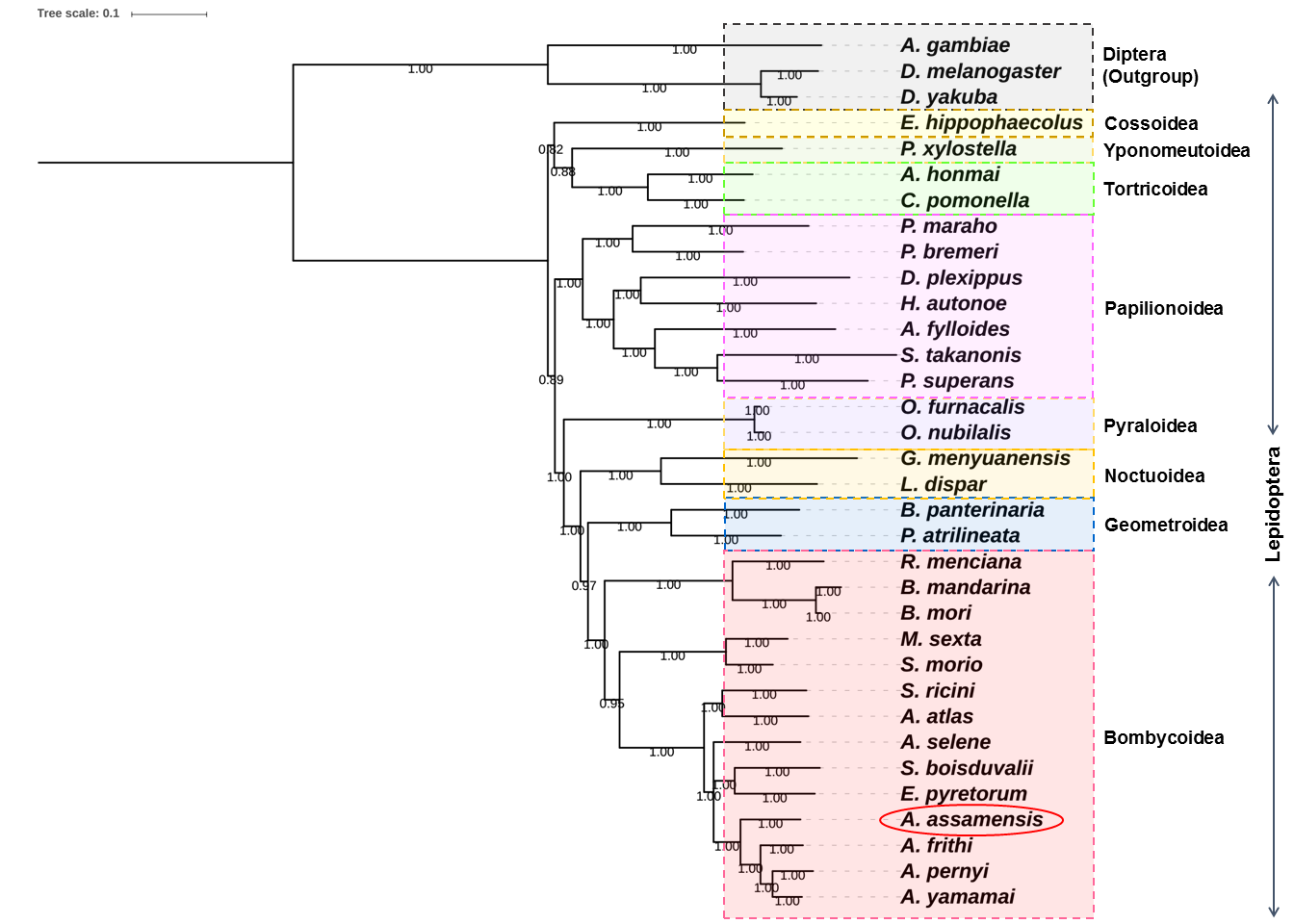

Supplement: S8 Fig — The tree is drawn to scale with bayesian posterior probability values indicated along with the branches. (TIF) [file pone.0188077.s008.tif]
