# Supplementary material for: The mitochondrial genome of Muga silkworm (Antheraea assamensis) and its comparative analysis with other lepidopteran insects
Source: PLoS One. 2017 Nov 15;12(11):e0188077. doi: 10.1371/journal.pone.0188077 (PMC5687760; doi:10.1371/journal.pone.0188077)
Supplement: S1 Table — (PDF) [file pone.0188077.s009.pdf]

S1 Table. Comparative gene lengths in Bombycoids.

Genes	*A. assamensis* (in bp)	*S. ricini* (in bp)	*A. pernyi* (in bp)	*A. yamamai* (in bp)	*B. mandarina* (in bp)	*B. mori* (in bp)	*M. sexta* (in bp)
tRNA^{Met}	**68**	68	67	67	68	68	69
tRNA^{Ile}	**65**	65	64	64	66	66	65
tRNA^{Gln}	**69**	69	69	69	69	69	69
nad2	**1014**	1014	1014	1014	1023	1023	1015
tRNA^{Trp}	**68**	68	68	68	70	70	67
tRNA^{Cys}	**64**	62	61	63	67	67	64
tRNA^{Tyr}	**67**	65	66	67	66	66	66
cox1	**1531**	1540	1531	1540	1531	1531	1531
tRNA^{Leu2}	**68**	68	66	67	67	67	67
cox2	**682**	685	682	682	682	682	682
tRNA^{Lys}	**71**	71	71	71	71	71	71
tRNA^{Asp}	**67**	68	73	69	67	67	72
atp8	**165**	165	168	186	162	162	165
atp6	**678**	678	677	677	678	678	678
cox3	**789**	789	789	789	789	789	792
tRNA^{Gly}	**66**	66	66	67	69	66	66
nad3	**357**	357	354	357	351	351	354
tRNA^{Ala}	**65**	66	66	66	68	68	70
tRNA^{Arg}	**65**	64	65	65	64	64	68
tRNA^{Asn}	**65**	65	65	65	67	65	66
tRNA^{Ser1}	**66**	68	66	66	67	67	66
tRNA^{Glu}	**68**	66	66	66	63	65	70
tRNA^{Phe}	**66**	68	71	69	67	67	68
nad5	**1741**	1749	1741	1744	1719	1719	1723
tRNA^{His}	**65**	66	69	66	69	67	69
nad4	**1341**	1341	1341	1341	1344	1341	1335
nad4l	**291**	291	291	292	291	291	288
tRNA^{Thr}	**65**	65	65	65	66	65	67
tRNA^{Pro}	**65**	65	65	65	69	66	65
nad6	**537**	531	537	535	531	531	531
cytb	**1149**	1152	1149	1155	1158	1152	1155
tRNA^{Ser2}	**66**	66	68	69	66	66	66
nad1	**933**	939	939	933	945	945	936
tRNA^{Leu1}	**68**	68	68	71	71	70	68
rrnL	**1344**	1358	1369	1380	1350	1378	1391
tRNA^{Val}	**68**	66	67	68	71	69	65
rrnS	**779**	779	775	776	784	783	775
**Control region**	**328**	361	552	334	484	494	326
**Whole mito-genome**	**15,272**	**15,384**	**15,575**	**15,338**	**15,682**	**15,656**	**15,516**

**Total PCGs**	**11,208**	**11,231**	**11,213**	**11,245**	**11,204**	**11,195**	**11,185**
**Total tRNAs**	**1,465**	**1,463**	**1,472**	**1,473**	**1,488**	**1,476**	**1,484**
**Total rRNAs**	**2,123**	**2,137**	**2,144**	**2,156**	**2,134**	**2,161**	**2,166**
