# Supplementary material for: The mitochondrial genome of Muga silkworm (Antheraea assamensis) and its comparative analysis with other lepidopteran insects
Source: PLoS One. 2017 Nov 15;12(11):e0188077. doi: 10.1371/journal.pone.0188077 (PMC5687760; doi:10.1371/journal.pone.0188077)
Supplement: S2 Table — C here denotes conserved sites, I denotes phylogenetically informative sites, V denotes variable sites, N denotes nucleotide level, A denotes amino acid level. The number of sites is represented in terms of percentage values. (PDF) [file pone.0188077.s010.pdf]

**S2 Table. Gene by gene divergences in the protein coding genes at different taxonomic levels, both at nucleotide level and amino acid levels.** C here denotes conserved sites, I denotes phylogenetically informative sites, V denotes variable sites, N denotes nucleotide level, A denotes amino acid level. The number of sites is represented in terms of percentage values.

| Sites | Hierarchy level | *nad2* | *cox1* | *cox2* | *atp8* | *atp6* | *cox3* | *nad3* | *nad5* | *nad4* | *nad4l* | *nad6* | *cytb* | *nad1* |
|---|---|---|---|---|---|---|---|---|---|---|---|---|---|---|
| **C-N** | **Species** | 84.1 | 88.9 | 89.2 | 86.3 | 87.8 | 87.1 | 90.5 | 90.1 | 89.9 | 90.5 | 83.1 | 85.3 | 86.3 |
| | **Genus** | 78.8 | 84 | 85 | 81.5 | 82.4 | 80.6 | 80.4 | 85.9 | 85.2 | 85 | 73.3 | 78.6 | 81.6 |
| | **Family** | 70.7 | 78.5 | 80 | 64.3 | 73.5 | 71.5 | 70.4 | 76.9 | 76.1 | 75.9 | 61.3 | 70.4 | 71 |
| | **Superfamily** | 66 | 76.1 | 78 | 56 | 71.3 | 68.6 | 67.9 | 72.6 | 70.8 | 73.8 | 56.1 | 66.4 | 68.1 |
| | **Order** | 51.2 | 68.5 | 69.2 | 41.7 | 62.5 | 60.7 | 58.4 | 59.7 | 59.7 | 58.2 | 41.9 | 58.1 | 59.5 |
| **V-N** | **Species** | 12.7 | 10.4 | 10.4 | 13.7 | 11.9 | 12.5 | 9.22 | 9.05 | 9.81 | 8.5 | 16.3 | 13.2 | 11.5 |
| | **Genus** | 17.9 | 15.2 | 14.6 | 18.5 | 17.4 | 19.1 | 19.3 | 13.4 | 14.5 | 13.9 | 26.1 | 20.1 | 16.9 |
| | **Family** | 27.2 | 20.7 | 19.6 | 35.7 | 26.2 | 28.2 | 29.3 | 22.6 | 23.6 | 24.1 | 38.7 | 28.9 | 28.1 |
| | **Superfamily** | 31.9 | 23.1 | 21.6 | 44 | 28.4 | 31.1 | 31.8 | 27.1 | 28.9 | 26.2 | 43.9 | 32.8 | 30.9 |
| | **Order** | 46.8 | 30.9 | 30.8 | 58.3 | 37.2 | 38.9 | 41.3 | 40 | 40 | 41.8 | 58.1 | 41.1 | 39.5 |
| **I-N** | **Species** | 0 | 0 | 0 | 0 | 0 | 0 | 0 | 0 | 0 | 0 | 0 | 0 | 0 |
| | **Genus** | 3.34 | 2.72 | 3.21 | 2.38 | 3.82 | 2.65 | 1.68 | 2.56 | 2.75 | 2.04 | 3.89 | 3.08 | 3.77 |
| | **Family** | 18.4 | 12.2 | 12.3 | 24.4 | 18.8 | 16.7 | 18.2 | 14.9 | 14.9 | 12.9 | 24.3 | 16.1 | 18.3 |
| | **Superfamily** | 20 | 14.2 | 13.3 | 28.6 | 20 | 18.6 | 19.6 | 16.5 | 16.1 | 15.3 | 27.4 | 18.2 | 19.4 |
| | **Order** | 22 | 15.9 | 15.8 | 33.3 | 22.5 | 21.7 | 23.5 | 18.5 | 19.3 | 19 | 32 | 21.5 | 21.5 |
| **C-A** | **Species** | 86 | 98.2 | 96.9 | 76.8 | 94.2 | 93.9 | 90.8 | 91.8 | 91.3 | 87.8 | 83.3 | 92.5 | 91.8 |
| | **Genus** | 81.1 | 97.9 | 94.3 | 69.6 | 92 | 90.9 | 80.7 | 88.5 | 87.5 | 81.6 | 74.4 | 88.4 | 86.2 |
| | **Family** | 66.5 | 91.1 | 89 | 42.9 | 78.3 | 80.3 | 68.9 | 75.2 | 72.5 | 67.3 | 55.6 | 78.4 | 67 |
| | **Superfamily** | 61 | 90.1 | 86.8 | 39.3 | 76.1 | 77.3 | 64.7 | 69.4 | 67 | 64.3 | 52.2 | 74.8 | 63.2 |
| | **Order** | 40.1 | 79.8 | 71.9 | 25 | 61.5 | 67.8 | 53.8 | 53.7 | 52.7 | 46.9 | 31.7 | 62 | 51.6 |
| **V-A** | **Species** | 10.6 | 1 | 2.63 | 21.4 | 5.31 | 5.3 | 8.4 | 7.4 | 8.26 | 10.2 | 15.6 | 5.66 | 5.7 |
| | **Genus** | 15.5 | 1.4 | 5.26 | 28.6 | 7.52 | 8.33 | 18.5 | 10.8 | 12.1 | 16.3 | 24.4 | 10 | 11.9 |
| | **Family** | 31.2 | 8.2 | 10.5 | 55.4 | 21.2 | 18.9 | 30.3 | 24.1 | 27 | 31.6 | 43.9 | 20.6 | 31.8 |
| | **Superfamily** | 36.7 | 9.1 | 12.7 | 58.9 | 23.5 | 22 | 34.5 | 30.1 | 32.6 | 34.7 | 47.2 | 24.2 | 35.5 |
| | **Order** | 57.9 | 19.6 | 28.1 | 73.2 | 38.1 | 31.4 | 45.4 | 45.8 | 46.9 | 52 | 67.8 | 37 | 47.2 |
| **I-A** | **Species** | 0 | 0 | 0 | 0 | 0 | 0 | 0 | 0 | 0 | 0 | 0 | 0 | 0 |
| | **Genus** | 2.9 | 0.2 | 1.32 | 1.79 | 2.65 | 1.14 | 0.84 | 2.6 | 2.01 | 1.02 | 1.7 | 1.8 | 1.3 |
| | **Family** | 24.4 | 6 | 6.14 | 37.5 | 18.6 | 13.6 | 22.7 | 19.8 | 19 | 20.4 | 33.3 | 11.6 | 20.4 |
| | **Superfamily** | 25.5 | 6.2 | 6.58 | 37.5 | 19 | 15.5 | 23.5 | 20.5 | 19.6 | 22.4 | 35 | 12.6 | 21.7 |
| | **Order** | 27.5 | 6.4 | 8.3 | 44.6 | 20.4 | 16.3 | 26.1 | 21.5 | 22.5 | 25.5 | 38.9 | 13.4 | 23.9 |
