# Supplementary material for: The mitochondrial genome of Muga silkworm (Antheraea assamensis) and its comparative analysis with other lepidopteran insects
Source: PLoS One. 2017 Nov 15;12(11):e0188077. doi: 10.1371/journal.pone.0188077 (PMC5687760; doi:10.1371/journal.pone.0188077)
Supplement: S3 Table — (PDF) [file pone.0188077.s011.pdf]

S3 Table. Anticodons (AC) of tRNAs and number of mismatches present in Amino-acyl (AA) stem, TΨC-stem, DHU-stem and Anticodon (AC) stem of mitochondrial tRNAs of *A. assamensis* with respect to the selected Bombycoids.

tRNA	Stem	*A. assamensis*	*S. ricini*	*A. pernyi*	*A. yamamai*	*B. mandarina*	*B. mori*	*M. sexta*
tRNA^{Met}	**AC**	**CAT**	CAT	CAT	CAT	CAT	CAT	CAT
	**AA Stem**	-	-	-	-	-	-	-
	**TΨC Stem**	-	-	-	-	-	-	-
	**AC Stem**	**GU-1**	**GU-1**	**GU-1**	**GU-1**	**GU-1**	**GU-1**	**GU-1**
	**DHU Stem**	-	-	-	-	-	-	-
tRNA^{Ile}	**AC**	**GAT**	GAT	GAT	GAT	GAT	GAT	GAT
	**AA Stem**	-	-	-	**GU-1**	-	-	-
	**TΨC Stem**	-	-	-	-	-	-	-
	**AC Stem**	**GU-1**	**GU-1**	**GU-1**	**GU-1**	-	-	**GU-1**
	**DHU Stem**	-	-	-	-	-	-	-
tRNA^{Gln}	**AC**	**TTG**	TTG	TTG	TTG	TTG	TTG	TTG
	**AA Stem**	-	-	-	-	-	-	-
	**TΨC Stem**	-	-	-	-	-	-	-
	**AC Stem**	-	-	-	-	-	-	-
	**DHU Stem**	**GU-1**	**GU-1**	**GU-1**	**GU-1**	-	**GU-1**	**GU-1**
tRNA^{Trp}	**AC**	**TCA**	TCA	TCA	TCA	TCA	TCA	TCA
	**AA Stem**	**GU-1**	**GU-1**	**GU-1**	**GU-1**	**GU-1**	**GU-1**	**GU-1**
	**TΨC Stem**	-	-	-	-	-	-	-
	**AC Stem**	**GU-1**	**GU-1**	**GU-1**	**GU-1**	-	-	-
	**DHU Stem**	-	-	-	-	-	-	-
tRNA^{Cys}	**AC**	**GCA**	GCA	GCA	GCA	GCA	GCA	GCA
	**AA Stem**	**GU-1**	**GU-1**	**GU-1**	**GU-2**	**GU-1**	**GU-1**	**GU-1**
	**TΨC Stem**	-	-	-	**AA-1**	-	-	-
	**AC Stem**	-	-	-	-	-	-	-
	**DHU Stem**	**GU-1**	**GU-1**	**GU-1**	-	-	-	-
tRNA^{Tyr}	**AC**	**GTA**	GTA	GTA	GTA	GTA	GTA	GTA
	**AA Stem**	-	-	-	-	-	-	-
	**TΨC Stem**	**GU-3, GA-1**	**AA-1**	**AA-1**	**AA-1**	-	-	-
	**AC Stem**	-	-	-	-	-	-	-
	**DHU Stem**	-	-	-	-	-	-	-
tRNA^{Leu2}	**AC**	**TAA**	TAA	TAA	TAA	TAA	TAA	TAA
	**AA Stem**	**GU-1, UU-1**	**UU-1**	**UU-1**	**UU-1**	**UU-1**	**UU-1**	**UU-1**
	**TΨC Stem**	-	-	-	-	-	-	-
	**AC Stem**	-	-	-	-	-	-	-
	**DHU Stem**	**GU-1**	**GU-1**	**GU-1**	**GU-1**	-	**GU-1**	**GU-1**
tRNA^{Lys}	**AC**	**CTT**	CTT	CTT	CTT	CTT	CTT	CTT
	**AA Stem**	-	-	-	-	-	-	-
	**TΨC Stem**	-	-	-	-	-	-	-
	**AC Stem**	-	-	-	-	-	-	-
	**DHU Stem**	-	-	-	-	-	-	-

tRNA^{Asp}	**AC**	**GTC**	GTC	GTC	GTC	GTC	GTC	GTC
	**AA Stem**	-	-	-	-	-	-	-
	**TΨC Stem**	-	-	-	-	-	-	-
	**AC Stem**	-	-	-	-	-	-	**GU-1**
	**DHU Stem**	-	-	-	-	-	-	-
tRNA^{Gly}	**AC**	**TCC**	TCC	TCC	TCC	TCC	TCC	TCC
	**AA Stem**	-	-	-	-	-	-	-
	**TΨC Stem**	-	-	-	-	-	-	-
	**AC Stem**	-	-	-	-	-	-	-
	**DHU Stem**	**GU-1**	**GU-1**	**GU-1**	**GU-1**	**GU-1**	**GU-1**	**GU-1**
tRNA^{Ala}	**AC**	**TGC**	TGC	TGC	TGC	TGC	TGC	TGC
	**AA Stem**	**GU-1, UU-1**	**GU-1, UU-1**	**GU-1, UU-1**	**GU-1, UU-1**	**GU-1**	**GU-1**	**GU-1, UU-1**
	**TΨC Stem**	-	-	-	-	-	-	-
	**AC Stem**	-	-	-	-	-	-	-
	**DHU Stem**	-	-	-	-	-	-	-
tRNA^{Arg}	**AC**	**TCG**	TCG	TCG	TCG	TCG	TCG	TCG
	**AA Stem**	-	-	-	-	-	**UU-1**	-
	**TΨC Stem**	-	-	-	-	-	**UU-1**	-
	**AC Stem**	-	-	-	-	-	-	-
	**DHU Stem**	-	-	-	-	-	-	-
tRNA^{Asn}	**AC**	**GTT**	GTT	GTT	GTT	GTT	GTT	GTT
	**AA Stem**	**GU-1**	-	-	-	-	-	-
	**TΨC Stem**	-	**AA-1**	**AA-1**	**AA-1**	-	-	-
	**AC Stem**	-	-	-	-	-	-	-
	**DHU Stem**	-	-	-	-	-	-	-
tRNA^{Ser1}	**AC**	**GCT**	GCT	GCT	GCT	GCT	GCT	GCT
	**AA Stem**	-	-	-	-	-	-	-
	**TΨC Stem**	**GU-1**	**GU-1**	**GU-1**	**GU-1**	**GU-1**	**GU-1**	**GU-1**
	**AC Stem**	-	-	-	-	-	-	-
	**DHU Stem**	**UU-1**	**UU-1**	**UU-1**	**UU-1**	-	-	**AA-1**
tRNA^{Glu}	**AC**	**TTC**	TTC	TTC	TTC	TTC	TTC	TTC
	**AA Stem**	**UU-1**	-	-	**GU-1**	-	-	-
	**TΨC Stem**	-	-	-	-	-	-	-
	**AC Stem**	-	-	-	-	-	-	-
	**DHU Stem**	-	-	-	-	-	-	-
tRNA^{Phe}	**AC**	**GAA**	GAA	GAA	GAA	GAA	GAA	GAA
	**AA Stem**	-	-	-	-	-	-	-
	**TΨC Stem**	-	-	-	**GU-1**	**GU-1, UU-1**	**GU-1, UU-1**	-
	**AC Stem**	-	-	-	-	-	-	-
	**DHU Stem**	**GU-1**	**GU-1**	**GU-1**	**GU-1**	**GU-2**	**GU-2**	**GU-2**
tRNA^{His}	**AC**	**GTG**	GTG	GTG	GTG	GTG	GTG	GTG
	**AA Stem**	**GU-1**	-	-	-	-	-	-
	**TΨC Stem**	-	**GU-1**	**GU-1**	-	**GU-1**	**GU-1**	-
	**AC Stem**	**GU-1**	**GU-1**	**GU-1**	-	**GU-1**	**GU-1**	**UU-1**

	DHU Stem	-	-	-	-	GU-1	GU-1	-
$tRNA^{Thr}$	AC	TGT	TGT	TGT	TGT	TGT	TGT	TGT
	AA Stem	-	GU-1, UU-1	-	-	-	-	-
	TΨC Stem	-	-	-	-	UU-1	UU-1	-
	AC Stem	GU-1	-	GU-1	GU-1	-	GU-1	GU-1
	DHU Stem	-	-	-	-	-	-	-
$tRNA^{Pro}$	AC	TGG	TGG	TGG	TGG	TGG	TGG	TGG
	AA Stem	-	-	-	-	-	-	-
	TΨC Stem	GU-1	GU-1	GU-1	-	-	-	-
	AC Stem	-	-	-	-	-	-	-
	DHU Stem	GU-1	GU-1	GU-1	GU-1	GU-1	GU-1	GU-1
$tRNA^{Ser2}$	AC	TGA	TGA	TGA	TGA	TGA	TGA	TGA
	AA Stem	-	-	-	-	-	-	-
	TΨC Stem	-	-	-	-	-	-	-
	AC Stem	UU-2	UU-2	UU-2	UU-2	UU-2	GU-1, UU-2	UU-1
	DHU Stem	-	-	-	-	-	-	-
$tRNA^{Leu1}$	AC	TAG	TAG	TAG	TAG	TAG	TAG	TAG
	AA Stem	GU-2	GU-2	GU-2	GU-1	GU-1	GU-1	UU-1
	TΨC Stem	-	GU-1	-	-	-	-	-
	AC Stem	GU-2	GU-1	GU-2	GU-2	GU-2	GU-2	GU-1
	DHU Stem	GU-1	GU-1	GU-1	GU-1	GU-1	GU-1	GU-1
$tRNA^{Val}$	AC	TAC	TAC	TAC	TAC	TAC	TAC	TAC
	AA Stem	-	GU-1	-	-	-	-	-
	TΨC Stem	-	-	-	-	-	-	-
	AC Stem	-	-	-	-	-	-	-
	DHU Stem	GU-1	GU-1	GU-1	GU-1	GU-1	GU-1	GU-1
GU Mismatches		28	24	23	22	18	22	17
UU Mismatches		6	6	5	5	5	7	5
GA Mismatches		1	2	2	3	0	0	0
AA Mismatches		0	0		0	0	0	1
Total Mismatches		35	32	30	30	23	29	23
