# Supplementary material for: The mitochondrial genome of Muga silkworm (Antheraea assamensis) and its comparative analysis with other lepidopteran insects
Source: PLoS One. 2017 Nov 15;12(11):e0188077. doi: 10.1371/journal.pone.0188077 (PMC5687760; doi:10.1371/journal.pone.0188077)
Supplement: S4 Table — (PDF) [file pone.0188077.s012.pdf]

S4 Table. Intergenic spacers (IGS) and overlapping sequence (OS) between different genes in *A. assamensis* with respect to the selected Bombycoid species.

Spacer	*A. assamensis*	*S. ricini*	*A. pernyi*	*A. yamamai*	*B. mandarina*	*B. mori*	*M. sexta*
tRNA^{Met} - tRNA^{Ile}	1	1	9	9	-2	-2	10
tRNA^{Ile} - tRNA^{Gln}	-3	-3	-3	-3	-3	-3	-3
tRNA^{Gln} - nad2	50	54	56	53	48	47	54
nad2 - tRNA^{Trp}	7	9	8	8	5	5	0
tRNA^{Trp} - tRNA^{Cys}	-8	-8	-8	-8	-8	-8	-8
tRNA^{Cys} - tRNA^{Tyr}	4	0	19	10	6	6	10
tRNA^{Tyr} – cox1	6	0	2	2	17	15	4
cox1- tRNA^{Leu2}	0	0	0	-5	0	0	0
tRNA^{Leu2}- cox2	0	0	0	0	0	0	0
cox2 - tRNA^{Lys}	0	0	0	0	0	0	0
tRNA^{Lys}- tRNA^{Asp}	17	33	23	16	-1	-1	21
tRNA^{Asp} - atp8	0	0	0	0	0	0	0
atp8 - atp6	-7	-7	-7	-7	-7	-7	-7
atp6 – cox3	-1	-1	0	-1	12	14	18
cox3 - tRNA^{Gly}	2	2	2	2	2	2	6
tRNA^{Gly} - nad3	-3	-3	0	-3	3	3	0
nad3 - tRNA^{Ala}	-1	-2	0	-2	53	54	20
tRNA^{Ala}- tRNA^{Arg}	0	24	-1	-1	50	31	1
tRNA^{Arg}- tRNA^{Asn}	0	26	0	0	0	2	50
tRNA^{Asn}-tRNA^{Ser1}	2	13	1	2	0	1	3
tRNA^{Ser1}-tRNA^{Glu}	0	3	1	1	10	17	0
tRNA^{Glu} -tRNA^{Phe}	11	-2	10	6	-1	-1	-2
tRNA^{Phe} - nad5	0	0	0	0	4	4	0
nad5 - tRNA^{His}	0	-3	0	-3	16	21	12
tRNA^{His} - nad4	4	1	9	12	59	56	0
nad4 - nad4l	1	3	5	3	-1	-1	84
nad4l - tRNA^{Thr}	7	12	7	5	4	4	13
tRNA^{Thr} -tRNA^{Pro}	0	0	0	0	-1	0	-1
tRNA^{Pro} - nad6	2	2	2	2	1	2	1
nad6 - cytb	2	7	2	-1	55	49	17
cytb - tRNA^{Ser2}	31	5	25	24	-1	3	21
tRNA^{Ser2} - nad1	23	24	19	24	25	24	31
nad1 - tRNA^{Leu1}	1	2	2	1	-6	-6	0
tRNA^{Leu1}- rrnL	0	0	0	0	33	0	0
rrnL - tRNA^{Val}	0	0	0	0	0	-1	0
tRNA^{Val} - rrnS	0	0	0	0	0	0	0
***rrnS*-control region**	0	0	0	0	0	0	0
**control region-*tRNA^{Met}***	0	0	0	0	0	0	0
**IGS regions**	**17**	**17**	**18**	**17**	**18**	**20**	**18**

**Total IGS basepairs**	**171**	**221**	**202**	**180**	**403**	**360**	**376**
**OS regions**	**6**	**8**	**4**	**10**	**10**	**9**	**5**
**Total OS basepairs**	**23**	**29**	**19**	**34**	**31**	**30**	**21**
