# Supplementary material for: The mitochondrial genome of Muga silkworm (Antheraea assamensis) and its comparative analysis with other lepidopteran insects
Source: PLoS One. 2017 Nov 15;12(11):e0188077. doi: 10.1371/journal.pone.0188077 (PMC5687760; doi:10.1371/journal.pone.0188077)
Supplement: S5 Table — (PDF) [file pone.0188077.s013.pdf]

S5 Table. AT skewness and GC skewness in the whole mitogenome sequence, protein coding genes (PCGs), tRNAs, rRNAs and control region of *A. assamensis* and the selected Bombycoid species.

Species Name	Whole mitogenome		Majority strand		Minority strand	
	AT Skew	GC Skew	AT Skew	GC Skew	AT Skew	GC Skew
A. assamensis	**-0.02**	**-0.22**	**-0.13**	**-0.13**	**-0.12**	**0.15**
S. ricini	-0.01	-0.23	-0.12	-0.13	-0.13	0.08
A. pernyi	-0.02	-0.22	-0.13	-0.13	-0.12	0.06
A. yamamai	-0.02	-0.22	-0.13	-0.13	-0.12	0.08
B. mandarina	0.06	-0.20	-0.04	-0.09	-0.15	0.08
B. mori	0.06	-0.22	-0.03	-0.12	-0.15	0.07
M. sexta	-0.01	-0.18	-0.10	-0.07	-0.13	0.08
**Species Name**	**PCGs**		**Majority strand PCGs**		**Minority strand PCGs**	
	**AT Skew**	**GC Skew**	**AT Skew**	**GC Skew**	**AT Skew**	**GC Skew**
A. assamensis	**-0.16**	**0.01**	**-0.14**	**-0.14**	**-0.19**	**0.32**
S. ricini	-0.16	0.02	-0.14	-0.15	-0.20	0.33
A. pernyi	-0.17	0.01	-0.15	-0.15	-0.19	0.31
A. yamamai	-0.16	0.02	-0.15	-0.15	-0.18	0.32
B. mandarina	-0.14	0.05	-0.05	-0.10	-0.27	0.31
B. mori	-0.13	0.03	-0.05	-0.13	-0.27	0.33
M. sexta	-0.14	0.05	-0.11	-0.09	-0.19	0.31
**Species Name**	**tRNA**		**Majority strand tRNAs**		**Minority strand tRNAs**	
	**AT Skew**	**GC Skew**	**AT Skew**	**GC Skew**	**AT Skew**	**GC Skew**
A. assamensis	**0.02**	**0.18**	**0.01**	**0.02**	**0.04**	**0.46**
S. ricini	0.01	-0.15	0.03	0.02	-0.02	-0.44
A. pernyi	0.00	-0.14	0.02	0.03	-0.03	-0.43
A. yamamai	0.01	-0.13	0.04	0.02	-0.04	-0.42
B. mandarina	0.03	-0.15	0.05	0.01	0.00	-0.44
B. mori	0.03	-0.15	0.05	0.02	0.00	-0.46
M. sexta	0.00	-0.11	0.01	0.03	-0.01	-0.38
**Species Name**	**rRNA**		***rrnL***		***rrnS***	
	**AT Skew**	**GC Skew**	**AT Skew**	**GC Skew**	**AT Skew**	**GC Skew**
A. assamensis	**-0.03**	**-0.36**	**-0.02**	**-0.37**	**-0.05**	**-0.34**
S. ricini	-0.02	-0.39	-0.01	-0.38	-0.04	-0.40
A. pernyi	-0.03	-0.39	-0.01	-0.40	-0.05	-0.37
A. yamamai	-0.03	-0.36	-0.02	-0.38	-0.05	-0.34
B. mandarina	0.03	-0.37	0.05	-0.39	0.01	-0.32
B. mori	0.03	-0.40	0.05	-0.40	0.00	-0.38
M. sexta	-0.03	-0.34	-0.03	-0.33	-0.04	-0.35

Species Name	Control Region	
	AT Skew	GC Skew
A. assamensis	**-0.10**	**-0.45**
S. ricini	-0.02	-0.21
A. pernyi	-0.09	-0.13
A. yamamai	-0.07	-0.31

B. mandarina	-0.02	-0.04
B. mori	-0.06	-0.27
M. sexta	-0.06	-0.33
